# A Modern Approach to Treat Molar/Incisor Pattern Periodontitis—Review

**DOI:** 10.3390/jcm12186107

**Published:** 2023-09-21

**Authors:** Taewan J. Kim, Caroline G. Littlejohn, Kristen H. Richey, Neda Falsafi, Chenshuang Li, Tun-Jan Wang, Bradley Lander, Yu-Cheng Chang

**Affiliations:** 1 Department of Periodontics, School of Dental Medicine, University of Pennsylvania, 240 S 40th Street, Philadelphia, PA 19104, USA; taewank@upenn.edu (T.J.K.); carlit@upenn.edu (C.G.L.); richeykh@upenn.edu (K.H.R.); nfalsafi@upenn.edu (N.F.); tjwang@upenn.edu (T.-J.W.); landerb@upenn.edu (B.L.); 2 Department of Orthodontics, School of Dental Medicine, University of Pennsylvania, 240 S 40th Street, Philadelphia, PA 19104, USA; lichens@upenn.edu

**Keywords:** localized aggressive periodontitis, molar–incisor pattern, guided tissue regeneration, host response modulation, antibiotics

## Abstract

Molar–incisor pattern periodontitis (MIPP) is a severe form of periodontal disease characterized by rapid attachment loss and bone destruction affecting the molars and incisors. Formerly referred to as aggressive periodontitis, the terminology for this condition was revised after the 2017 workshop on the classification of periodontal and peri-implant diseases and conditions. Despite the modification in nomenclature, the treatment strategies for MIPP remain a critical area of investigation. The core principles of MIPP treatment involve controlling local and systemic risk factors, managing inflammation, and arresting disease progression. Traditional non-surgical periodontal therapy, including scaling and root planing, is commonly employed as an initial step together with the prescription of antibiotics. Surgical intervention may be necessary to address the severe attachment loss. Surgical techniques like resective and regenerative procedures can aid in achieving periodontal health and improving esthetic outcomes. This review article aims to provide an overview of the current understanding and advancements in the treatment modalities of MIPP. Through an extensive analysis of the existing literature, we discuss various modern therapeutic approaches that have been explored for managing this challenging periodontal condition.

## 1. Introduction

The most recent classification of aggressive periodontitis was outlined by the 1999 American Academy of Periodontology (AAP) Committee on the Classification of Periodontal Disease. This committee described two forms of periodontitis: chronic (slowly progressing) and aggressive (rapidly progressing) disease [1]. Aggressive periodontitis was first described as a “diffuse alveolar atrophy” by Gottlieb in 1923, calling the disease “periodontosis”. This concept and term were supported by Orban in 1924. However, in the 1966 American Academy of Periodontology World Workshop in Periodontics, “periodontosis” was not considered its own entity. In 1969, Chaput introduced the term “juvenile periodontitis”, a phrase later defined by Baer as “a disease of the periodontium occurring in an otherwise healthy adolescent which is characterized by a rapid loss of alveolar bone about more than one tooth of the permanent dentition” [2].

In the 1999 classification, aggressive periodontitis was characterized by three primary features: rapid loss of attachment, an otherwise healthy subject, and familial aggregation. In addition to those criteria, this classification sought to delineate between aggressive and chronic periodontitis. While aggressive periodontitis generally displays rapid progression with a strong familial aggregation, chronic periodontitis has a relatively slow rate of progression and is less associated with familial aggregation [2].

As outlined in the Consensus report of workgroup 2 of the 2017 Proceedings of the World Workshop on the Classification of Periodontal and Peri-Implant Diseases and Conditions, updated knowledge from the last 30 years supports three forms of periodontitis: periodontitis, necrotizing periodontitis, and periodontitis as a manifestation of systemic disease. “Chronic” and “aggressive” forms of the disease were combined into a single category—periodontitis—and gradients of the disease can be described through the staging and grading system outlined in the Consensus report [3].

However, according to the 2017 World Workshop’s Classification of Aggressive Periodontal Disease review paper, there are considerable factors that support aggressive periodontitis as its own entity, which include the aggressive nature of the disease, location of the lesions, familial tendencies, and the minimal amount of subgingival biofilm [2]. The idea of localized versus generalized aggressive periodontitis was discussed as well, as it was proposed that generalized aggressive periodontitis is not a separate entity from the localized form but rather a progression of the initial, localized disease [2]. It was proposed by Gunsolley et al. that the aggressive form of disease moved from localized to generalized if serum IgG or IgA levels were not effective against the responsible pathogens, like *Aggregatibacter actinomycetemcomitans* (*Aa*), leading to their overgrowth over time [3]. This highlights the importance of the host response not only in periodontal disease but especially in the aggressive form of the disease, as those with an adequate host antibody response to infectious agents do not progress from the localized to generalized form of the disease [3].

**Figure 1:**
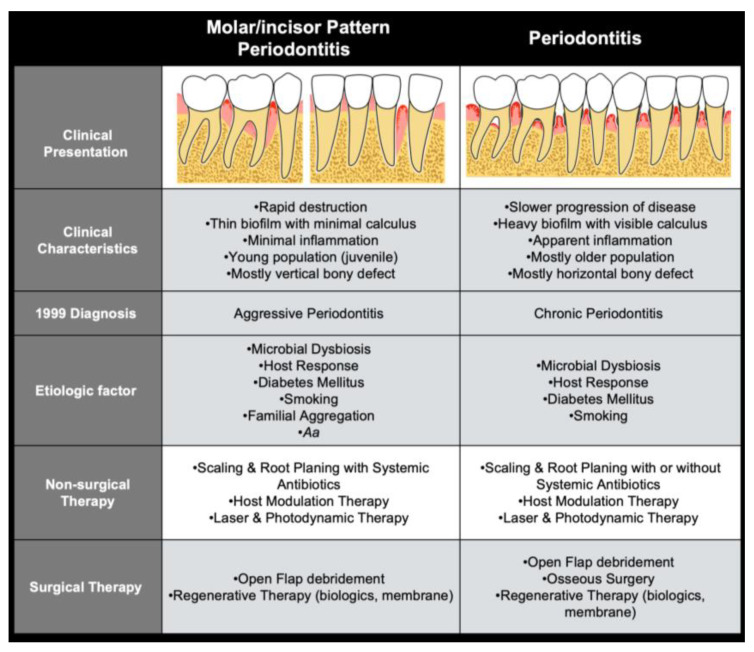
Summary of etiology and treatment modalities with updated classification of periodontitis.

In this workshop, a new idea for a staging system for aggressive periodontitis was discussed, combining age, location, and the extent of disease. The definitions of periodontal disease are dynamic, and although “aggressive periodontitis” is not a separate entity of the current classification, an argument was made for a future reclassification of this disease, so that with improved categorization, there can be a better understanding of the pathologic process [2]. Changes in the diagnosis for aggressive periodontitis might create confusion, but the approach for the treatment is still the same as they are based on the severity and risk factors (Figure 1). Therefore, the goal of this review is to summarize the traditional modalities (adjunctive antibiotic therapy and flap surgery) and provide the most updated evidence for the common current modalities (guided tissue regeneration with or without biologics) and the future modalities (laser/photodynamic therapy and host response modulation) that will open new avenues for the clinician and patient.

## 2. Adjunctive Antibiotic Therapy

Conventional scaling and root planing (SRP) is generally believed to be a successful approach for a reduction in microbial count [4]. However, this treatment method does not eliminate the microorganisms from anatomic areas that are difficult to access, including furcation areas, infrabony defects, the tongue, and tonsils. The administration of systemic antimicrobials is widely used in the treatment of periodontal disease to eliminate periodontal infections caused by *Aggregatibacter actinomycetemcomitans* (*Aa*) and other periodontal pathogens that are part of the red complex (*T. forsythia*, *P. gingivalis*, and *T. Denticola*), which invade subepithelial tissues and are difficult to eradicate with non-surgical therapy alone. Because MIPP is characterized by thin biofilm and hyperresponsive immune reaction, and therefore, eradicating pathogens is highly effective with these patients. There is a consensus that mechanical debridement is the first therapeutic phase to reduce the subgingival bacterial load and the use of systemic antibiotics has been widely used and researched as an adjunct in the treatment of molar–incisor pattern periodontitis due to specific pathogens were believed to have a strong, causative relationship with the disease (Table 1).

It is worth mentioning that systemic antibiotics should only be considered as a supportive treatment, as mechanical debridement is still considered an essential element when treating periodontal disease. In 1998, Berglundh et al. separated patients with advanced periodontitis into four categories: Antibiotics Alone, Antibiotics + SRP, No Treatment, and SRP Alone [5]. They recorded the clinical parameters and found that antibiotic therapy alone was not as effective as SRP alone at improving clinical parameters such as reducing probing depth (PD), increasing clinical attachment level (CAL), and decreasing the percentage of bleeding on probing (BOP), illustrating that systemic antibiotics themselves are not a replacement for classic initial phase therapy: oral hygiene instruction (OHI) and SRP [6]. However, the best outcomes were seen in the group that received both antibiotics and SRP, wherein the mean probing depth was reduced from about 7.9 mm to 4.8 mm, and the attachment loss decreased from 8.1 mm to 6.4 mm at 36 months, illustrating the importance of antibiotics as an adjunct to SRP in the treatment of periodontitis [6].

**Table 1:** Summary studies using adjunctive systemic antibiotics in patients with molar–incisor pattern periodontitis.

Study Author (Year)	Type of Patient	Treatment Modality	Antibiotic Regimen	Duration of Study	Total Number of Patients	Treatment Groups	Number of Patients per Group	Clinical Data	Baseline	After Baseline		
										2–4 Months	6 Months	12 Months
Berglundh (1998) [6]	Advanced Periodontal Disease	Antibiotics or Placebo, SRP or No Mechanical Therapy	Amoxicillin: 375 mg BID for 14 days; Metronidazole: 250 mg TID for 14 days	2 Years	16	Antibiotic Therapy + No Scaling	8 Quadrants	PD (mm)	4.6 ± 0.6	3.9 ± 0.4	-	3.6 ± 0.3
								CAL (mm)	-	0.2 ± 0.2	-	0.2 ± 0.2
								BOP (%)	65 ± 15	54 ± 12	-	51 ± 11
								Plaque (%)	72 ± 5	9 ± 4	-	13 ± 5
						Antibiotic Therapy + Scaling	8 Quadrants	PD (mm)	4.8 ± 0.7	3.2 ± 0.3	-	3.1 ± 0.3
								CAL (mm)	-	0.6 ± 0.4	-	0.8 ± 0.4
								BOP (%)	72 ± 15	20 ± 7	-	17 ± 6
								Plaque (%)	70 ± 4	10 ± 4	-	10 ± 4
						Placebo + No Scaling	8 Quadrants	PD (mm)	4.8 ± 0.8	4.5 ± 0.8	-	4.5 ± 1.0
								CAL (mm)	-	−0.1 ± 0.1	-	−0.3 ± 0.3
								BOP (%)	70 ± 7	65 ± 6	-	69 ± 11
								Plaque (%)	68 ± 6	9 ± 5	-	11 ± 6
						Placebo + SRP	8 Quadrants	PD (mm)	4.5 ± 0.8	3.2 ± 0.4	-	3.1 ± 0.4
								CAL (mm)	-	0.6 ± 0.2	-	0.7 ± 0.3
								BOP (%)	71 ± 8	19 ± 5	-	24 ± 12
								Plaque (%)	68 ± 5	9 ± 5	-	10 ± 4
Guerrero (2005) [7]	Generalized Aggressive Periodontitis (GAP)	SRP and Chlorhexidine Rinse, Systemic Antibiotics in Experiemental Group	Amoxicillin: 500 mg TID for 7 days; Metronidazole: 500 mg TID for 7 days	6 Months	41	SRP + Chlorhexidine Rinse	21	PD (mm)	4.1	3.3	3.4	-
								CAL (mm)	4.8	4.3	4.3	-
								BOP (%)	55	38	34	-
								Plaque (%)	20	27	20	-
						SRP + Chlorhexidine Rinse + Antibiotic Therapy	20	PD (mm)	4.1	3	2.9	-
								CAL (mm)	4.7	4	3.9	-
								BOP (%)	61.5	27	29.5	-
								Plaque (%)	25.5	19.5	24.5	-
Xajigeorgioue (2006) [8]	Generalized Aggressive Periodontitis (GAP)	Scaling and Root Planing, Scaling 6 Weeks Later, Systemic Antibiotics for Experimental Groups	Amoxicillin: 500 mg TID for 7 days; Metronidazole: 500 mg TID for 7 days; Doxycycline: 200 mg Loading Dose, 100 mg QD for 14 days	6 Months	43	Control	11	PD (mm)	4.2 ± 0.7	-	3.5 ± 0.8	-
								CAL (mm)	4.6 ± 0.7	-	4.1 ± 0.6	-
								BOP (%)	78 ± 37	-	15 ± 25	-
						Metronidazole + Amoxicillin	10	PD (mm)	4.6 ± 1.0	-	3.1 ± 0.7	-
								CAL (mm)	5.0 ± 1.0	-	4.0 ± 1.3	-
								BOP (%)	87 ± 21	-	15 ± 14	-
						Doxycycline	10	PD (mm)	4.2 ± 0.6	-	3.4 ± 0.8	-
								CAL (mm)	5.0 ± 1.4	-	4.2 ± 1.9	-
								BOP (%)	81 ± 25	-	14 ± 22	-
						Metronidazole	12	PD (mm)	4.7 ± 0.6	-	2.9 ± 0.6	-
								CAL (mm)	5.4 ± 1.3	-	4.1 ± 1.3	-
								BOP (%)	80 ± 36	-	21 ± 31	-
Haas (2008) [9]	Generalized Aggressive Periodontitis (GAP)	Scaling and Root Planing, Systemic Antibiotics	Azithromycin: 500 mg/day for 3 days	12 Months	24	Placebo	12	PD (mm)	4.7 ± 1.9	-	-	2.85 ± 0.36
								CAL (mm)	3.1 ± 2.5	-	-	4.07 ± 0.29
								BOP (%)	23 ± 22	-	-	44.46 ± 3.89
								Plaque (%)	72 ± 29	-	-	-
						Azithromycin (1500 mg/day)	12	PD (mm)	4.8 ± 2.1	-	-	1.92 ± 0.23
								CAL (mm)	3.5 ± 2.6	-	-	5.18 ± 0.20
								BOP (%)	15 ± 19	-	-	45.04 ± 3.32
								Plaque (%)	56 ± 33	-	-	-
Machtei (2008) [10]	Aggressive Periodontitis	SRP, Systemic Antibiotics	Amoxicillin: 500 mg TID for 7 days; Metronidazole: 500 mg TID for 7 days; Doxycycline: 200 mg Loading Dose, 100 mg QD for 14 days	3 Months	31	Doxycycline	15	PD (mm)	4.09 ± 0.1	3.37 ± 0.1	-	-
								CAL (mm)	4.93 ± 0.3	4.02 ± 0.2	-	-
								GI	1.58 ± 0.2	0.37 ± 0.1	-	-
								PI	1.43 ± 0.2	0.31 ± 0.1	-	-
						Metronidazole + Amoxicillin	14	PD (mm)	4.29 ± 0.2	3.53 ± 0.2	-	-
								CAL (mm)	4.93 ± 0.3	4.14 ± 0.2	-	-
								GI	2.02 ± 0.2	0.35 ± 0.1	-	-
								PI	1.78 ± 0.2	0.30 ± 0.1	-	-
Mestnik (2010) [11]	Generalized Aggressive Periodontitis (GAP)	SRP and Chlorhexidine Rinse, Systemic Antibiotics in Experimental Group	Amoxicillin: 500 mg TID day for 14 days; Metronidazole: 400 mg TID day for 14 days	3 Months	30	Placebo	15	PD (mm)	4.1 ± 0.6	3.2 ± 0.6	-	-
								CAL (mm)	4.2 ± 0.5	3.5 ± 0.5	-	-
								BOP (%)	63.6 ± 21.3	12.5 ± 11.7	-	-
						Amoxicillin + Metronidazole	15	PD (mm)	4.3 ± 0.7	2.7 ± 0.5	-	-
								CAL (mm)	4.5 ± 0.8	3.2 ± 0.5	-	-
								BOP (%)	77.7 ± 19.7	12.2 ± 13.0	-	-
Yek (2010) [12]	Generalized Aggressive Periodontitis (GAP)	SRP, Systemic Antibiotics in Experimental Group	Amoxicillin: 500 mg TID for 7 days; Metronidazole: 500 TID for 7 days	6 Months	28	Placebo	16	PD (mm)	3.7 ± 0.7	2.5 ± 0.5	2.5 ± 0.5	-
								CAL (mm)	3.3 ± 1.3	2.30 ± 1.16	2.4 ± 1.1	-
						Amoxicillin + Metronidazole	12	PD (mm)	4.06 ± 0.6	2.7 ± 0.5	2.6 ± 0.4	-
								CAL (mm)	3.8 ± 1.1	2.7 ± 1.1	2.8 ± 1.3	-
Baltacioglu (2011) [13]	Generalized Aggressive Periodontitis (GAP)	SRP, Systemic Antibiotics in Experimental Group	Amoxicillin: 250 mg TID for 10 days; Metronidazole: 250 mg TID for 10 days; Doxycycline: 200 mg Loading Dose, 100 mg QD for 14 days	2 Months	36	SRP Only	12	PD (mm)	4.93 ± 0.31	4.21 ± 0.19	-	-
								CAL (mm)	5.49 ± 0.45	4.70 ± 0.61	-	-
								BOP (%)	95.00 ± 0.10	37.70 ± 0.12	-	-
						SRP + Amoxicillin + Metronidazole	14	PD (mm)	4.86 ± 0.74	3.37 ± 0.39	-	-
								CAL (mm)	5.28 ± 0.81	3.91 ± 0.51	-	-
								BOP (%)	93.57 ± 0.11	25.21 ± 0.13	-	-
						SRP + Doxycycline	12	PD (mm)	4.96 ± 0.62	3.96 ± 0.34	-	-
								CAL (mm)	5.68 ± 0.81	4.63 ± 0.43	-	-
								BOP (%)	95.17 ± 0.11	36.58 ± 0.17	-	-
Heller (2011) [14]	Generalized Aggressive Periodontitis (GAP)	SRP, Systemic Antibiotics in Experiemental Group	Metronidazole: 250 mg TID for 10 days; Amoxicillin: 500 mg TID for 10 days	6 Months	31	SRP Only	15	PD (mm)	4.9 ± 0.2	3.5 ± 0.2	3.5 ± 0.2	-
								CAL (mm)	5.2 ± 0.2	4.4 ± 0.2	4.4 ± 0.2	-
								BOP (%)	83.6 ± 4.4	54.0 ± 6.4	69.0 ± 5.3	-
						SRP + Amoxicillin + Metronidazole	16	PD (mm)	5.2 ± 0.2	3.3 ± 0.1	3.2 ± 0.1	-
								CAL (mm)	5.6 ± 0.3	4.1 ± 0.2	4.1 ± 0.3	-
								BOP (%)	85.0 ± 3.1	45.0 ± 3.7	60.0 ± 4.7	-
Varela (2011) [15]	Generalized Aggressive Periodontitis (GAP)	SRP, Systemic Antibiotics in Experimental Group	Amoxicillin: 500 mg TID for 10 days; Metronidazole: 250 mg TID for 10 days	6 Months	31	SRP Only	15	PD (mm)	4.2 ± 0.2	3.3 ± 0.1	3.3 ± 0.1	-
								CAL (mm)	4.6 ± 0.3	4.0 ± 0.2	3.9 ± 0.2	-
								BOP (%)	81 ± 4.9	50.9 ± 3.8	57.9 ± 4.9	-
						SRP + Amoxicillin + Metronidazole	16	PD (mm)	4.3 ± 0.2	3.1 ± 0.1	2.9 ± 0.1	-
								CAL (mm)	4.9 ± 0.3	3.8 ± 0.2	3.8 ± 0.2	-
								BOP (%)	85.7 ± 3.6	41.4 ± 2.7	45.1 ± 4.2	-
Merchant (2014) [16]	Localized Aggressive Periodontitis (LAP)	Scaling and Root Planing, Systemic Antibiotics	Amoxicillin: 500 mg TID for 7 days; Metronidazole: 250 mg TID for 7 days	12 Months	97	Primary Dentition	22	PD (mm)	4.84 ± 0.65	3.00 ± 0.81	2.85 ± 0.84	2.59 ± 1.15
								CAL (mm)	3.47 ± 1.44	0.32 ± 0.34	0.51 ± 0.56	0.43 ± 0.64
								BOP (%)	3.47 ± 1.45	8.90 ± 4.93	12.10 ± 9.67	7.85 ± 4.39
								Plaque (%)	3.47 ± 1.46	25.50 ± 17.43	35.30 ± 25.91	38.85 ± 13.22
						Permanent Dentition	75	PD (mm)	3.47 ± 1.47	3.71 ± 1.01	3.82 ± 0.86	3.65 ± 0.90
								CAL (mm)	3.47 ± 1.49	1.45 ± 1.40	1.60 ± 1.67	1.26 ± 1.56
								BOP (%)	3.47 ± 1.50	12.20 ± 9.07	0.39 ± 8.96	13.86 ± 16.25
								Plaque (%)	3.47 ± 1.51	27.02 ± 17.77	30.57 ± 23.63	29.54 ± 15.17
Taiete (2016) [17]	Generalized Aggressive Periodontitis (GAP)	OHI, SRP, Systemic Antibiotics OR Placebo	Amoxicillin: 375 mg TID for 7 days; Metronidazole: 250 mg TID for 7 days	6 Months	39	SRP + Placebo	18	PD (mm)	6.4 ± 0.4	-	4.3 ± 0.7	-
								CAL (mm)	8.6 ± 1.0	-	7.1 ± 0.9	-
								BOP (%)	32.6 ± 7.6	-	12.5 ± 5.5	-
								Plaque (%)	38.2 ± 6.8	-	24.4 ± 3.9	-
						SRP + Amoxicillin + Metronidazole	21	PD (mm)	6.5 ± 0.5	-	3.8 ± 0.8	-
								CAL (mm)	8.6 ± 1.4	-	10.3 ± 0.9	-
								BOP (%)	35.6 ± 6.7	-	11.5 ± 3.8	-
								Plaque (%)	28.5 ± 11.1	-	22.5 ± 5.1	-
Lu (2021) [18]	Generalized Aggressive Periodontitis (GAP)	Scaling and Root Planing, Systemic Antibiotics for Experimental Group	Amoxicillin: 0.5 g TID; Metronidazole: 0.2 g TID for 7 days	6 Months	42	SRP Only + Placebo	14	PD (mm)	3.47 ± 1.52	-	3.0 ± 0.3	-
						Amoxicillin + Metronidazole After SRP	14	PD (mm)	5.4 ± 1.0	-	2.8 ± 0.5	-
						Amoxicillin + Metronidazole During SRP	14	PD (mm)	5.1 ± 0.7	-	2.7 ± 0.3	-

More recently, Miller evaluated the long-term (4 years) clinical response to periodontal therapy and maintenance in localized aggressive periodontitis (LAP) patients. In this study, all 124 patients underwent SRP and a regimen of amoxicillin (AMOX) + metronidazole (MET) [19]. The cohort displayed significant reductions in PD, attachment loss, percentage of deep and bleeding sites, percentage of sites affected by LAP, and percentage of plaque when compared to the baseline [19]. Yek’s study from 2010 utilizes a control group (SRP only) and an experimental group (AMOX + MET), finding significantly more reduction in PD and significantly more attachment gain in the experimental group (*p* < 0.05) [12]. These findings are further supported by Jiao, who recorded outcomes of SRP + OHI and SRP + OHI + AMOX + MET for 1004 patients with GAP [12]. After initial phase therapy, analysis showed that additional PD reduction was found when the antibiotic therapy was administered in addition to SRP and OHI [12]. The difference in probing depth for sites with initial PD ≥ 5 mm was 0.26 mm, favoring the administration of systemic antibiotics (*p* < 0.001) [20]. The effectiveness of MET + AMOX for deep periodontal pockets is supported by Guerrero’s findings, in which patients receiving SRP + MET + AMOX had an additional 1.4 mm of probing depth reduction and 1 mm of attachment gain at 6 months for pockets ≥ 7 mm compared to SRP alone. In total, 74% of the pockets with PD ≥ 5 mm at baseline were 4 mm or shallower at 6 months in the test group, compared with 54% in the control group (*p* = 0.008) [7]. Additional findings supporting the use of adjunctive antibiotics with initial phase therapy are abundant, but not all studies on the topic agree that the use of systemic antibiotics makes a significant difference [11,21]. Varela studied the effects of AMOX + MET+ SRP compared to SRP alone for patients with GAP and found that although both groups exhibited improved parameters such as PD reductions and increased CAL, there was not a significant difference between the two therapeutic groups at 6 months [15]. Additionally, a systemic review by Mendes studying AMOX + MET + SRP found that antibiotics did not significantly improve the CAL, although their administration did lead to a statistically significant difference in probing depth between the two groups, the mean difference being 0.40 mm (*p* = 0.02), favoring MET + AMOX [22].

The administration of antibiotics has significant effects on the microbiology of the periodontal pocket. Mestnik studied the changes in bacterial populations by comparing SRP alone versus SRP + MET+ AMOX and found counts of pathogens making up the red complex were more significantly reduced in the SRP + AMOX + MET group (2.8%) in comparison with the SRP group (8.1%). Regarding *Aa*, the antibiotics group displayed a significant reduction in the levels of *Aa* in initially deep sites compared with the control group (*p <* 0.05) [11]. Heller also studied the effects of antibiotic therapy on the subgingival microbiota in patients with GAP but found that the effects of AMOX + MET in addition to SRP was comparable to SRP in that most of the periodontal pathogens, like *Aa, P. gingivalis*, *T. forsythia*, *C. rectus*, and *P. micra*, decreased significantly in both groups, while the host-compatible species increased along with the “non-periodontal species” [14]. Yek also found that patients who were prescribed AMOX + MET had a continuous decrease in *T. denticola* over 6 months, whereas patients who only had SRP had no changes beyond 3 months [12].

The most widely studied combination of antibiotics in the treatment of molar–incisor pattern periodontitis is AMOX + MET [4]. In a study by Baltacioglu, three treatment modalities were explored: SRP + MET + AMOX, SRP + Doxycycline (DOX), and SRP Alone. All groups had improved periodontal index values, although the groups receiving systemic antibiotics had better improved probing depths and clinical attachment level values when compared to those who received SRP alone (*p* < 0.05). Additionally, probing depths were significantly improved for those receiving AMOX + MET compared to those who received SRP + DOX (*p* < 0.05) [13]. These results are similar to those found by Xajigeorgiou, who compared different antibiotic therapies—AMOX + MET, MET Alone, DOX, and SRP Alone—finding that the intake of MET alone and AMOX + MET led to a significant reductions in sites that were >6 mm compared with the control group (SRP Alone) [8]. However, Machtei studied the clinical and immunological parameters affected by SRP + DOX versus SRP + AMOX + MET, finding that both treatment protocols resulted in a significant improvement in the clinical and immunological parameters, but the differences between the treatment groups were not significant, save for PGE2, which was less for the DOX group than the AMOX + MET group. PGE2 levels in gingival crevicular fluid can be related to the rate of attachment loss, so the decreased levels of this cytokine are regarded as an improved parameter [10]. Taiete found that the administration of AMOX + MET led to improved probing depths compared to SRP alone and a statistically significant reduction in PGE2 in deep pockets at both 3 and 6 months after therapy (*p* < 0.05). [17] In addition, the administration MET + AMOX was the only treatment that resulted in statistically significant reductions in levels of *P. gingivalis*, *T. forsythia*, *T. denticola*, and *Aa*, a reduction that was maintained until 6 months from the baseline [8]. From the overwhelming evidence supporting the use of combination antibiotic therapy (AMOX + MET), questions arise about the antibiotic of choice in patients with penicillin allergies. Haas studied the use of azithromycin as an adjunct to SRP compared to SRP + placebo. In this study, both treatment modalities led to significantly improved plaque scores and significantly less BOP. Azithromycin led to a significantly higher reduction in mean PD of about 1 mm compared to the placebo (*p* = 0.025) and more improvement in CAL than the control group (*p* = 0.05), making this a viable alternative in penicillin-allergic patients [9].

Due to its rapidly progressive nature, the timing of the administration of systemic antibiotics becomes an important decision in clinical care. Beliveau compared the clinical outcome of a systemic antibiotic regimen prescribed immediately after or 3 months following mechanical therapy for patients with LAP using AMOX + MET [23]. When comparing the reductions in probing depth and gains in attachment level 3 months after each group started antibiotic therapy, no differences were observed between the groups (*p* > 0.05) [23]. Recently, Lu et al. reported similar findings when they studied the clinical outcomes for patients with GAP who received SRP only and those who were prescribed AMOX + MET during or after SRP. They found that PD reduction was positively related to the adjunctive use of antibiotics, regardless of timing (2.7 ± 0.9 mm and 2.5 ± 0.7 mm versus 1.8 ± 0.8 mm, *p* < 0.05). These results were most prominently seen in sites with deeper initial PD sites and sites with infrabony defects [18]. These results imply that antibiotics are effective, no matter the timing, but without a benefit of starting later, there is no reason to delay therapy. The severity of the disease seems to play more of a role in PD reduction, similar to our findings when treating chronic periodontitis.

In addition, the dosage of the prescribed antibiotic regimens could affect the outcomes of therapy. In a systematic review and meta-analysis by Karrabi et al., the efficacy of the dose and duration of AMOX + MET administration in the treatment of Stage II–III Grade C periodontitis was evaluated. Since the dose of AMOX was generally the same in all included studies in the review, doses of MET were compared with one another, more specifically 250 mg vs. 400–500 mg. They found a significant difference in PD reduction in moderate pockets treated with 250 mg MET versus 400–500 mg, favoring the higher dosage [24]. When looking at severe pockets treated with antibiotics, there was no significant reduction in pocket depth for patients treated with 250 mg MET, but there was a significant difference compared to the control when patients were treated with 400–500 mg MET (the probing depth mean difference being 1.32 mm) [24]. This difference in test groups was significant, as was the reduction in probing depths for those treated with 400–500 mg in the severe pockets. [24] The effect of the 250 mg dosage compared to the baseline was insignificant, implying that the 250 mg MET dose was not effective in eliminating pathogens in cases of moderate to severe attachment loss, supporting an argument for the use of a higher dosage of MET [24]. When reflecting on Varela’s findings that were discussed previously, where there was no difference in clinical parameters for those treated with adjunct antibiotics, it is important to keep in mind that they utilized a 250 mg dose of MET, which falls at the lower end of the dosage range and may be the reason there were no significant differences between SRP alone and SRP + AMOX + MET [15]. Patient compliance plays a role in regard to the outcomes of clinical parameters. There was significantly more reduction in PD and a greater CAL increase for compliant patients. Noncompliance with the antibiotic and maintenance regimen reduced the amount of improvement for these patients [19,25].

Antibiotics are routinely administered in conjunction with mechanical debridement to treat MIPP, effectively reducing the microbial load in periodontal pockets, particularly bacteria in the red complex and *Aa* [4,21]. Numerous studies have shown that adding antibiotics improves clinical parameters such as reduced probing depths, increased clinical attachment, and microbial population improvement in periodontal pockets [4,21]. The combination of AMOX + MET is the most extensively researched and evidence-based choice, with azithromycin as a viable alternative for penicillin-allergic individuals [9]. Administering antibiotics immediately after mechanical debridement is preferred to avoid delaying treatment benefits [23]. This review recommends a dosage of 500 mg AMOX and 500 mg MET for moderate to severe pockets. Systemic antibiotics enhance the effectiveness of initial phase therapy by reducing microbial load and eliminating etiological factors, potentially reducing the need for surgical interventions. The strategy of prescribing the antibiotics when treating MIPP should be similar with other extents and distributions (generalized and localized periodontitis).

## 3. Flap Surgery

**Figure 2:**
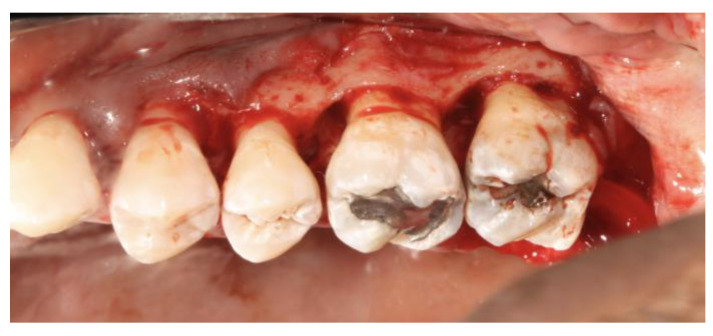
Representation of open flap debridement for molar–incisor pattern periodontitis.

A classic surgical intervention for patients with molar–incisor pattern periodontitis is the use of an access flap and debridement of the root surface as represented in Figure 2. As discussed previously, the 2017 World Workshop removed aggressive periodontitis from its classification, instead combining aggressive and chronic periodontitis in the general category of periodontitis. A 1984 paper by Lindhe compared the outcomes of adult and juvenile periodontitis patients with angular bony defects adjacent to the first molars and incisors, a pattern that would be defined by the 2017 World Workshop as “Molar/Incisor Pattern”. Both groups were treated with an administration of tetracycline and open flap debridement (OFD) utilizing a modified Widman flap (MWF), and both groups demonstrated a similar, sizable magnitude of improvement in BOP and plaque index [26]. At the follow-up visits, there was a marked gain in clinical attachment for both groups, an improvement that lasted even 6 months after therapy. The average gain of clinical attachment in the molar regions of the juvenile patients ranged from 4.7 mm to 5.3 mm, and in the incisor regions, varied between 2.1 mm and 2.6 mm. For adult periodontitis patients, the clinical attachment gain was less pronounced: ranging from 3.0 mm to 3.4 mm in molar sites and from 1.8 mm to 2.2 mm in incisors. The similar response from the two groups to the therapy implies that different diseases can be treated with the same treatment with significant improvement [27].

Sewon compared radiographic bone fill after initial therapy and the utilization of an MWF for 10 patients. At the start of the study, the mean radiographic bone loss was 31.5%, which decreased to 23.7% by 12 months after surgical treatment was rendered. The amount of radiographic bone loss was 7.8% less than at the initial examination. At the 1-year mark, there were no new bone resorption sites, and there was either bone fill or a clear cessation of bone destruction. In addition, plaque and gingival indices showed substantial improvement at 1 year and at the final examination (plaque index: 0.16 ± 0.13% and gingival index: 0.02 ± 0.04%) [28]. In 2002, Buchman followed 13 patients for 5 years after SRP, OFD utilizing an MWF, and antibiotic therapy. Buchman recorded a gain in attachment of 2.23 ± 0.91 mm at 3 months, with an ultimate gain in attachment of 2.57 ± 1.06 mm at 5 years, demonstrating the effectiveness of OFD in addition to SRP and the administration of antibiotics [29]. Further supporting the use of surgical therapy, a case report from 2016 described a patient with aggressive periodontitis who was treated with an MWF procedure and connective tissue graft with a 2.5-year follow-up. The results demonstrated decreased probing depths, an improvement in marginal bone levels, and decreased levels of periodontal pathogens, demonstrating the usefulness of surgical intervention in the treatment of patients with aggressive periodontal disease [30]. Another case report in 2020 also details the utilization of OFD and root resection in the treatment of a 43-year-old man with Stage III Grade C Periodontitis. In the 2 years of suppurative therapy, the periodontal condition remained stable, and the bacterial load was reduced [31].

Although surgical intervention can lead to promising results, evidence appears to be inconclusive when comparing the effectiveness of SRP alone versus SRP with OFD. In a split-mouth randomized control trial by Wennström, which compared SRP alone with SRP + MWF, 16 patients were followed up to 5 years after treatment [27]. After treatment, both surgical and non-surgical sites showed improved plaque scores and decreased BOP. At the baseline, the mean values of probing pocket depth ranged from 6.5 mm to 7.4 mm. After treatment, for all therapy groups, the reduction amounted to 3–4 mm, with no remarkable difference in clinical parameters noted between the SRP and the SRP + MWF group. Probing attachment levels improved on average between 2.0 mm and 2.9 mm, with improvement being similar between the four treatment groups. Additionally, when analyzing the radiographs of teeth affected in the juvenile periodontitis group 6 months after therapy, teeth treated with surgery in the juvenile groups showed a 1.75 mm decrease in the distance between the cementoenamel junction (CEJ) and alveolar bone crest at the affected site compared to the baseline (*p* < 0.01), and the sites treated by SRP alone showed a 1.35 mm decrease from the baseline (*p* < 0.05). Excision of the granulation tissue in conjunction with flap elevation did not enhance the degree of probing pocket depth reduction, probing attachment gain, and bone fill following meticulous root surface instrumentation [27]. Christersson compared the outcomes of SRP alone, SRP + MWF, and SRP + soft tissue curettage over the course of 16 weeks, finding a substantial decrease in plaque and gingival inflammation scores in all groups. The mean probing depth for SRP did not significantly improve or worsen throughout the study. Meanwhile, the probing depths for the treatment groups that received surgical intervention showed statistically significant decreases in probing depths at both 8 and 16 weeks. Concerning the clinical attachment level, at 16 weeks, the groups receiving surgical intervention had significantly more attachment gain when compared to the group that received SRP alone [32]. More recently, Cirino compared the outcomes of SRP alone compared to those obtained with OFD. In this study, clinical parameters such as bleeding on probing and plaque index were significantly decreased after therapy, regardless of modality. In addition, both treatment modalities promoted a significant reduction in probing depths and gain in clinical attachment levels (*p* < 0.05). However, there was no significant difference found between treatment modalities when comparing all initial probing depths. When the pockets were delineated between moderate and deep, the deep pockets treated with surgical therapy had a significantly greater decrease in probing depth (4.8 ± 0.6 mm) compared to those treated with non-surgical therapy (5.9 ± 1.2 mm) [33]. Overall, evidence suggests that surgical intervention as an adjunct to SRP may provide additional benefits in terms of probing depth reduction and clinical attachment gain in aggressive periodontitis.

In 1976, Newman et al. and Slots et al. found an association between *Aa* and aggressive periodontitis [34,35]. Christersson’s 1985 study, which compared SRP, SRP + MWF, and SRP + soft tissue curettage, studied improvements in clinical parameters and the microbiological makeup of the subgingival sites. This study found that SRP did not markedly affect the subgingival *Aa* counts. For the surgical groups, the number of sites with *Aa* dramatically decreased. There was a positive correlation between the loss of clinical attachment and the presence of *Aa* 16 weeks post treatment [32]. All of the periodontal lesions that showed a loss of attachment contained *Aa*, and 57% of the lesions that displayed no change in attachment level harbored *Aa*. Of the sites that gained clinical attachment, only 22% contained *Aa*. The relationship between *Aa* and aggressive periodontitis has already been established, but these results imply that SRP alone is not sufficient for the elimination of this bacteria and thus not sufficient for the treatment of aggressive periodontitis [32]. In 1988, Mandell found that the combination of surgery and DOX led to decreased levels of *Aa* at affected sites compared to the baseline, a decrease that lasted the duration of the 12-month study concerning clinical parameters; bleeding on probing and redness were significantly (*p* < 0.001 and *p* < 0.02, respectively) reduced from the baseline to 12 months. In addition, pocket depths were significantly reduced (*p* < 0.01), and attachment level was significantly improved (*p* < 0.02). There was only one patient in the study who followed the 3-month maintenance program, and for this patient, no *Aa* was detectable at 12 months, emphasizing the importance of continued maintenance after surgical therapy [36]. However, a more recent study by Cirino in 2019 compared non-surgical and surgical therapies through a split-mouth design and found no difference in the levels of *Aa* or *P. gingivalis* between the two treatment groups at any time point up to 12 months [33].

After reviewing the literature regarding surgical interventions in the treatment of molar–incisor pattern periodontitis, there is evidence that OFD is a viable treatment with an improvement in clinical parameters and elimination of pathogens in periodontal pockets (Table 2). However, there is no overwhelming evidence that surgical intervention is superior to SRP, whether or not antibiotics are used as adjuncts. Although OFD showed similar results as SRP, this is not to say that surgical intervention is not necessary, as the breadth of treatment modalities utilized in 2023 goes beyond OFD and will be discussed later in this review.

## 4. Guided Tissue Regeneration (GTR)

**Figure 3:**
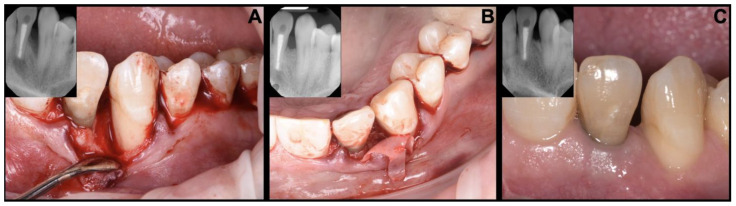
Representation of guided tissue regeneration with a barrier membrane for molar–incisor pattern periodontitis: (**A**) flap is raised exposing the defects, (**B**) placement of bone graft with barrier membrane, (**C**) healing post 6 months.

Guided tissue regeneration (GTR) is a regenerative therapy that is commonly used for treating deep pockets and periodontal defects in patients with periodontitis [37]. Guided tissue regeneration typically consists of opening a flap in the defect and then placing a membrane with or without a bone graft to prevent epithelial migration into the site of the defect to allow the slower populating bone growth cells into the area as clinically represented in Figure 3. It is believed that guided tissue regeneration is a treatment modality that can be used to treat aggressive periodontitis, especially the presence of deep and narrow defects.

Studies by Rakmanee et al. have compared guided tissue regeneration (GTR) to open flap debridement (OFD) in the treatment of aggressive periodontitis patients. Overall, the outcomes between GTR and other surgical procedures, including OFD and simplified papilla preservation flap (SPPF), did not show significant differences, although both approaches demonstrated a significant improvement compared to the baseline [37,38]. One split-mouth randomized controlled clinical trial examined the outcomes of performing GTR using a resorbable polyglycolide membrane versus an access flap with a simplified papilla preservation flap. Both methods resulted in improvements in clinical attachment levels and probing depths, with no statistically significant differences between the two approaches [37]. Another study by Rakmanee compared defects treated with a simplified papilla preservation flap to those treated with a GTR membrane. Both treatments showed significant improvements and a resolution of the defects. The GTR sites demonstrated enhanced bone fill and defect resolution at 12 months compared to the 6-month outcomes, highlighting the importance of evaluating long-term outcomes beyond 12 months [38].

Other studies compared using bone grafting material versus a membrane to see which one had a more favorable outcome in aggressive periodontitis patients. Mengel et al. in 2006 compared the use of a membrane versus bioactive glass for the treatment of defects in aggressive periodontitis patients. Both the membrane group and grafting group with bioactive glass showed improvements in probing depth and clinical attachment level after 5 years, but radiographically, the lesions treated with bioactive glass were more significantly filled [39]. Queiroz et al. in 2013 performed a split-mouth study where 15 patients with two defects were treated either with ABM-P-15, a synthetic bone graft, or GTR. They found that both significantly improved the clinical outcomes, but the sites treated with AMB-P-15 had better radiographic bone fill. In both studies, the clinical outcomes improved with both modalities, but the grafted groups had more radiographic bone fill [40]. Gorski et al. in 2020 compared a xenogeneic graft plus a modified perforated membrane to a xenogeneic graft plus a standard collagen membrane and found no statistically significant differences between the two membranes but both showed clinical benefits for maintaining compromised teeth for up to 4 years after surgery [41].

In addition to the beforementioned studies, several systematic reviews and meta-analyses have been completed to examine the best outcomes in the treatment of aggressive periodontitis with GTR [4,42]. Peterson et al. completed a meta-analysis that compared open flap debridement (OFD) with GTR. Meta-analyses showed that after 6 months, probing depth was, on average, reduced by 1 extra millimeter when using GTR than when using OFD, but there were no statistically significant differences at 12 months, suggesting that outcomes should be examined at least 12 months to see the true differences between the treatment modalities [43]. Diaz-Faes et al., in a meta-analysis of six clinical trials, showed that the use of biomaterials for regenerative therapy may be more effective than surgical debridement alone when looking at the parameters of probing depth and the distance between the CEJ and alveolar crest at 6 months but noted that more studies with a larger sample size and longer follow-up are needed [44].

Overall, guided tissue regeneration appears to be an effective approach for treating lesions in molar–incisor pattern periodontitis and demonstrates improvements in clinical parameters compared to no treatment. It can be concluded that evaluating outcomes at 12 months provides a more accurate assessment of the lasting results of GTR therapy. While GTR offers advantages, it yields comparable results to alternative treatments such as grafting, the application of biologics, and open flap debridement (Table 3).

## 5. Guided Tissue Regeneration with Biologics

**Figure 4:**
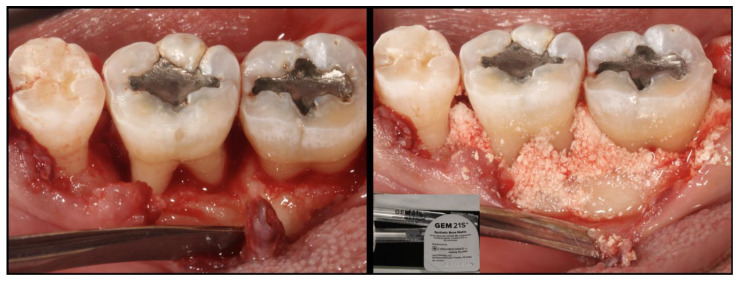
Clinical photos and radiographs showing the representation of guided tissue regeneration by the placement of a bone graft and barrier membrane.

Biologics are therapeutic agents with biologic activity and have been used frequently together with the regenerative approach. Some examples of biologics that have been used to treat periodontal disease are enamel matrix derivative (EMD), platelet-rich plasma (PRP), and platelet-rich fibrin (PRF). According to the American Academy of Periodontology’s best evidence consensus statement on the use of biologics in clinical practice, the use of biologics may significantly enhance the clinical and radiographic outcomes of infrabony defects. In addition, combination therapies involving bone grafts are the most effective strategies for treating infrabony defects [55]. As MIPP-related infrabony defects are mostly vertical and containable, the use of biologics is a suitable treatment option as represented in Figure 4.

Enamel matrix derivative (EMD) is a porcine product that consists of hydrophobic enamel matrix proteins from embryonal enamel. Amelogenin is the main constituent in EMD [56]. Several studies have used EMD to treat aggressive periodontitis and have demonstrated that that the use of EMD, when combined with GTR, yields a good result but were not able to show that EMD enhanced the outcomes over bone grafting alone [47,48,49]. Artzi et al., 2019 examined the outcomes of aggressive periodontal patients who had GTR or a demineralized bone xenograft (DBX) with EMD with a follow-up from 1–10 years. The two different methods both resulted in periodontal regeneration with a significant reduction in probing depths and clinical attachment loss in both groups, but there were no statistical differences between the two groups. The study emphasized the importance of meticulous surgical execution, strict maintenance, and patient compliance to achieve good results [49]. Kaner et al., 2008 described a case report of the treatment of a 27-year-old with probing depths of 7–12 mm in the molar region but no incisor involvement. The combination of initial therapy, antibiotics, minimally invasive flap surgery, and EMD showed successful 12-month results and provided a good clinical, radiographic, and esthetic result for the patient [47]. Artzi et al., 2015 studied aggressive periodontitis patients with multiple intrabony defects. EMD and GTR or GTR alone were the two treatment modalities used. Both treatment modalities showed similarly successful clinical results at 2 years post treatment [48].

It is debatable whether EMD can be used without bone grafting materials in order to achieve the same result. In a case study by Milauskaite et al., sites treated with EMD alone versus EMD with bioactive glass both had successful outcomes [46]. In this case report, a 19-year-old with localized aggressive periodontitis who was a non-smoker was treated with initial therapy, systemic antibiotics, a papilla preservation flap, and EMD in some sites and EMD with bioactive glass in other sites. The sites with EMD and EMD with bioactive glass both resulted in the successful treatment of intrabony defects [46]. 

When surgical debridement with EMD was compared with surgical debridement alone, there was no added benefit of using EMD [45]. Vandana et al. analyzed defects in both chronic and aggressive periodontitis patients and compared surgical debridement versus surgical debridement with EMD. It was performed as a split-mouth study, and the results showed that there was no advantage of using Emdogain when compared to surgical debridement alone. Both the Emdogain group and the surgical debridement group resulted in a decrease in pocket depth and attachment gain, which are indirect measures of the amount of regeneration occurring at the sites [45].

In contrast, Mazzonetto et al. showed that the use of EMD with conservative surgery showed additional benefits to conservative surgery alone [50]. Mazzonetto et al. studied the difference in outcomes of infrabony defects treated in both aggressive periodontitis patients and chronic periodontitis patients. These patients were treated with either conservative surgery or conservative surgery with EMD. Probing depth, clinical attachment level, and gingival recession were measured. This study found that the use of EMD promotes additional clinical benefits in the regeneration of infrabony defects in aggressive periodontitis, leading to a greater CAL gain and PPD reduction compared to a surgical approach alone. Moreover, EMD therapy led to similar clinical gains in patients with different diagnoses, aggressive or chronic periodontitis, except for a higher gingival recession in CP patients. This study also looked at oral-health-related quality-of-life outcomes estimated using OHIP-14. It was possible to observe a highly significant improvement in the quality of life for all patients after 6 months, regardless of the therapy, but root hypersensitivity was the most commonly found adverse effect and was more common in patients with aggressive periodontitis. This study suggests that the use of EMD may be a relevant therapeutic option for the treatment of aggressive periodontitis. Overall, the use of EMD may be a relevant therapeutic option for the treatment of molar–incisor pattern periodontitis, particularly in conjunction with appropriate surgical techniques and bone grafting materials [50].

Platelet-rich plasma (PRP) and platelet-rich fibrin (PRF) are naturally occurring growth factors that are concentrated by the clinician to create a platelet gel. The use of PRP as an adjunct to bone grafting was first introduced by Marx and Whitman [57,58]. To utilize PRP and/or PRF, the patient’s own blood is collected via venipuncture, spun down with a centrifuge, and polymerized. Platelets in the blood promote the initial coagulation and release growth factors that aid healing. In addition to containing growth factors, platelet-rich plasma also contains fibrinogen, fibronectin, and vitronectin. These proteins aid in cell adhesion and osteoconduction and make the platelet-rich plasma sticky, promoting hemostasis, stabilization, and immobilization of the graft. In addition, fibrin also adheres to the root surface to promote the epithelial migration of cells when the flap is re-adhering to the root surface. There are limited studies about the use of PRP and PRF to treat molar–incisor pattern periodontitis, but the studies that have been conducted have shown favorable outcomes when PRP have been used in addition to a bone graft or when PRF was used in addition to a flap procedure [51,52,53,54]. Gupta et al. conducted a controlled clinical trial treating aggressive periodontitis patients with bilateral intrabony defects. The patients were treated either with a hydroxyapatite graft or PRP with a hydroxyapatite graft. Both treatments provided significant improvements in clinical and radiographic parameters in a 12-month postoperative period. The PRP/HA group presented superior results regarding PD reduction, clinical attachment gain, and radiographic bone fill than the group that was treated with an HA graft alone [52].

PRF has been used as a therapy to treat molar–incisor pattern periodontitis. Thorat et al. in 2017 in a split-mouth study with 15 patients with localized aggressive periodontitis had two sites treated, one with a modified flap operation or a Kirkland flap and one with a Kirkland flap with PRF. This study found that the use of PRF significantly enhanced both the clinical and radiographic outcomes of open flap debridement, resulting in a decrease in probing depth and an increase in clinical attachment level [53]. In another study, fifty-four defects in 17 patients were treated either with autologous PRF with open flap debridement (OFD) or OFD alone. This study demonstrated that there was greater bone fill at sites treated with PRF than with only OFD, suggesting that PRF does help improve the outcome of patients with aggressive periodontitis [54].

There are limited studies about the use of GEM21, recombinant human-platelet-derived growth factor (rhPDGF-BB) with an osteoconductive matrix (β-TCP), as a treatment modality for patients with aggressive periodontitis. In addition, there is limited evidence about the use of specific growth factors as a treatment modality in the treatment of defects in patients with aggressive periodontitis. Corbella et al. in 2016 examined 22 full texts in a systematic review. Enamel matrix derivative (EMD) was used in six studies, bioactive glass (BG) in three studies, and bovine bone (BB) in five studies [59]. Other biomaterials were used in the included studies with or without the application of a resorbable or non-resorbable membrane. The reported outcomes did not show a significant advantage of one approach over the other [59]. Overall, the current literature does not show that EMD offers additional benefits to the treatment of defects in aggressive periodontitis, but some literature suggests the use of PRP and PRF in addition to surgery has showed superior outcomes to surgery alone. Some of the parameters that improved when using PRP or PRF were increased CAL, decreased PD, gain in radiographic bone fill, gain in gingival margin level, and decrease in IBD depth. More studies with larger sample sizes should be conducted to prove these results. In addition, there is limited evidence that compares the use of PRP and PRF to only non-surgical treatment. Further research is needed to determine the optimal protocols and long-term effectiveness of biologics in treating aggressive periodontitis (Table 3).

## 6. Laser and Photodynamic Therapy (aPDT)

**Figure 5:**
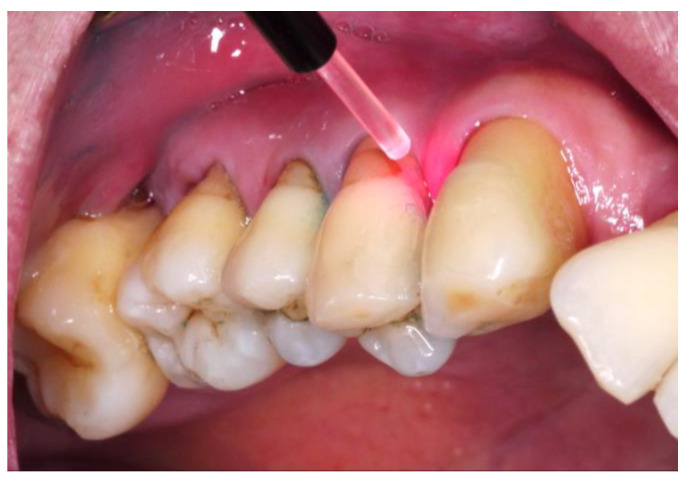
Representation of antimicrobial photodynamic therapy for molar–incisor pattern periodontitis. Photographs are provided by courtesy of Dr. Pang-Ning Chuang.

Treatment options for treating MIPP include traditional mechanical non-surgical and surgical approaches, often accompanied by additional anti-infective therapies involving disinfectants and antibiotics, as discussed earlier [60]. However, chemotherapeutic treatment has drawbacks such as the potential development of bacterial resistance and systemic side effects. Furthermore, non-surgical debridement has limitations in removing pathogens due to the complex anatomical structure of the tooth and the shape of the defect [61]. To overcome these limitations, laser and photodynamic therapies have been developed, which involve the use of laser irradiation with or without photosensitizer dye as represented in Figure 5 [62].

Various types of dental lasers have been employed for treating periodontal disease [63]. The specific laser used depends on factors such as power, wavelength, frequency, and properties. Different wavelengths can be used in combination with mechanical non-surgical techniques to remove pocket epithelium, inactivate bacteria, and eliminate subgingival calculus [64,65]. Another laser-based treatment method is antimicrobial photodynamic therapy (aPDT). In the context of diseased pockets, aPDT is applied to eliminate bacteria by generating reactive oxygen radicals through a combination of laser light and photosensitizer [60]. Additionally, aPDT offers the advantage of influencing photobiomdulation, reducing excessive inflammation caused by pathogens and irritation, and promoting photocoagulation. It can also stimulate the formation of blood clots and facilitate the healing process. Although laser-related treatments show promise in various aspects, for them to be recommended as a reliable treatment modality, they must consistently deliver predictable and safe outcomes that are superior to or at least equivalent to those achieved with conventional methods [63].

Matarese et al. demonstrated that the combination of scaling and root planing (SRP) with a diode laser resulted in a significant improvement in clinical parameters (probing depth (PD): 3.36 ± 0.51 mm vs. 2.56 ± 0.44 mm, clinical attachment level (CAL): 4.23 ± 0.21 mm vs. 3.44 ± 0.28 mm), while no notable differences were observed in microbial and inflammatory mediator levels. Kamma et al. found that a diode laser used in conjunction with SRP (PD: 3.87 ± 0.92 mm, CAL: 4.93 ± 1.67 mm) had a superior effect compared to SRP alone (PD: 4.13 ± 1.06 mm, CAL: 5.2 ± 1.66 mm) or laser treatment alone (PD: 3.93 ± 1.39 mm, CAL: 4.93 ± 1.67 mm) in terms of antimicrobial effect and clinical parameters when monitoring patients with aggressive periodontitis over a 6-month period [66]. Additionally, Mummolo et al. conducted a comparison between laser treatment alone and a surgical approach (open flap debridement), revealing similar outcomes and demonstrating the efficacy of laser treatment as being on par with surgery but without the associated disadvantages [67].

While multiple studies have shown the advantages of laser treatment over conventional SRP and even its similarity to surgical modalities, studies related to aPDT have not yielded clear results. Oliveira analyzed clinical parameters after treatment with aPDT alone compared to SRP, revealing that aPDT alone is as effective as SRP (aPDT − PD: 3.49 ± 0.98 mm, CAL: 8.74 ± 2.12 mm vs. SRP − PD: 3.98 ± 1.76 mm, CAL: 9.1 ± 3.05 mm) [61]. Al-Khureif et al. compared aPDT + SRP with systemic antibiotics + SRP, and aPDT showed a significant improvement in deep pockets (≥7 mm) (aPDT + SRP – PD: 3.82 ± 0.98 mm, CAL: 3.94 ± 1.01 mm vs. antibiotics + SRP − PD: 4.85 ± 0.85 mm, CAL: 5.75 ± 0.56 mm), while antibiotics + SRP was more effective in reducing proinflammatory cytokines [68]. These studies support the idea that laser treatment is superior in overcoming anatomical difficulties due to limited access but may not be as effective as antibiotics in reducing inflammation.

However, Borekci’s study did not demonstrate any advantage of aPDT + SRP over SRP alone, except for bleeding on probing (BOP), with a similar antimicrobial effect [69]. Furthermore, Arweiler et al. compared aPDT + SRP with antibiotics + SRP and found that clinical parameters were superior with antibiotic adjunctive treatment, suggesting that aPDT cannot replace the use of antibiotics [70]. The main difference between these two studies was the number of sessions of aPDT treatment. While Al-Khureif et al. applied aPDT on days 3, 7, and 14, Borekci et al. administered it twice (once after SRP and once the following day) [68,69]. These comparable results highlight the clear need for an effective protocol when utilizing aPDT as a treatment option.

As mentioned, there are different types of lasers used in dental treatment. Talmac et al. compared the diode laser with the Er,Cr:YSGG laser. Both laser applications demonstrated benefits in terms of clinical periodontal parameters and levels of gingival-crevicular-fluid cytokines when used in conjunction with SRP. Among the two types of lasers, the Er,Cr:YSGG laser exhibited a more successful outcome for aggressive periodontitis (Er,Cr:YSGG 0.91 mm vs. Diode 0.71 mm) [71]. Annaji et al. combined a laser and aPDT (with one or multiple sessions) and showed superior outcomes in both clinical parameters and pathogen removal (reduction in culture population) at 3 months after SRP + laser + aPDT with multiple sessions [72].

In summary, laser and aPDT therapies have demonstrated their efficacy and effectiveness compared to traditional treatment modalities. However, some studies have highlighted their limitations. Conducting thorough trials with larger sample sizes will be crucial, but the observed limitations, as deduced from various studies, mainly arise from the lack of a standardized protocol. With further investigations and the increased utilization of lasers and aPDT in clinical practice, the protocol will become more refined, allowing for effective utilization as an adjunctive or standalone modality, thus reducing the reliance on antibiotics and surgery (Table 4).

## 7. Host Response Modulation

Studies in molecular and cellular immunology have elucidated the pathogenesis of periodontitis, revealing that while biofilm serves as the primary etiological factor, the disease arises from complex interactions between dysbiotic bacterial pathogens and the host immune response, leading to uncontrolled inflammation [2]. Notably, molar–incisor pattern periodontitis exhibits an exacerbated immune response [76]. Therefore, alongside effective management of the active sites, controlling the host response becomes crucial for maintaining a healthy periodontium.

Shaddox et al. investigated the hypersensitivity of the immune response, a critical aspect of aggressive periodontitis, by studying familial aggregation. The objective of their study was to determine if a hyperresponsive trait, as seen in TLR-stimulated peripheral blood leukocytes, was present in a cohort of African Americans (the highest prevalent racial group) diagnosed with localized aggressive periodontitis (LAP) when compared to periodontally healthy related siblings and unrelated healthy participants [76]. As expected, the LAP group exhibited a hyperresponsive trait when proinflammatory responses were compared to those of unrelated and healthy individuals. The most surprising finding was that when patients with LAP were compared to their healthy siblings, the healthy siblings also displayed a robust cytokine response, although less pronounced than that of patients with LAP. This strongly suggests a genetic linkage of this “hyperresponsive” phenotype, and now-healthy siblings need to be closely monitored during maintenance, as they may develop an attenuated hyperinflammatory state that could lead to LAP [76].

Only a few studies have aimed to target the hyperresponsive host response. Azoubel et al. utilized Etoricoxib, a COX-2 inhibitor, but it did not provide additional improvement in clinical parameters. However, Etoricoxib did lead to an initial reduction in prostaglandin E2 (PGE2) levels in the gingival crevicular fluid (GCF), which could be associated with a discrete improvement in the bone condition [77]. Another pharmacological agent widely studied for chronic periodontitis is sub-antimicrobial dose doxycycline, which acts as an antibiotic with host modulation mechanisms [78]. It effectively inhibits matrix metalloproteinase activity, a key enzyme involved in periodontal breakdown. Furthermore, the sub-antimicrobial dose (20 mg twice a day) is not associated with antibiotic resistance development and has minimal side effects, making it safe for long-term use compared to other pharmacological agents [78]. Although it has shown efficacy in many studies compared to a placebo, no studies have specifically investigated its effects on molar–incisor pattern periodontitis. Therefore, future studies can explore its potential as a confirmed host modulation modality.

## 8. Conclusions

(1) Antibiotic treatment and surgical interventions: antibiotic administration plays a crucial role in the treatment of molar–incisor pattern periodontitis, effectively reducing the microbial load in periodontal pockets and improving clinical parameters. The combination of AMOX + MET remains the most researched and evidence-based choice, while azithromycin serves as an alternative for patients with penicillin allergies. Timely administration of antibiotics after mechanical debridement is recommended to maximize treatment benefits. Systemic antibiotics enhance the effectiveness of initial phase therapy, helping to achieve microbial reduction and eliminate etiological factors, potentially reducing the need for surgical interventions. Surgical interventions such as access surgery have shown promising results in improving clinical parameters and eliminating dysbiosis in periodontal pockets. However, there is no overwhelming evidence supporting its superiority over SRP, with or without adjunctive antibiotics.

(2) Guided tissue regeneration: limited evidence exists regarding the use of GEM21^TM^ and specific growth factors as treatment modalities for aggressive periodontitis. EMD does not appear to offer additional benefits for defect treatment; meanwhile, the use of PRP and PRF in addition to surgery has shown superior outcomes. More extensive studies are needed to validate these results, especially in comparison to non-surgical treatments. Conventional GTR has proven effective in treating aggressive periodontitis lesions and improving clinical parameters. A 12-month follow-up is recommended to assess long-term outcomes. GTR yields comparable results to grafting and open flap debridement.

(3) Future modalities—Laser and host response modulation: lasers and aPDT have demonstrated efficacy comparable to traditional treatment modalities. However, standardized protocols are lacking, leading to limitations in some studies. Further research with larger sample sizes is necessary to refine the protocols, enabling their effective use as adjunctive or standalone modalities, potentially reducing reliance on antibiotics and surgery. Lastly, host response modulation agents hold promise for future maintenance and preventative modalities by modulating the host response. This approach can extend beyond aggressive periodontitis and be applied to various inflammatory diseases, paving the way for new treatment directions. 

Last but not least, the term “aggressive periodontitis” was omitted because extensive similarities exist between aggressive periodontitis and chronic periodontitis upon comparison. Insufficient evidence supports the differentiation between these diseases. Our review article indicates that molar–incisor pattern periodontitis responds equally well to modern treatment approaches as general or localized periodontitis. The treatment outcome does not appear to be influenced by the molar–incisor pattern. This suggests that the effective control of plaque and the host immune response remains crucial in treating periodontitis. However, further randomized clinical trials are necessary to validate these observations.

## Figures and Tables

**Table 2:** Summary studies using open flap debridement (surgical approach) in patients with molar–incisor pattern periodontitis.

Study Author (Year)	Type of Patient	Treatment Modality	Antibiotic Regimen	Duration of Study	Total Number of Patients	Treatment Groups	Number of Patients per Group	Clinical Data	Baseline	After Baseline		
										2–4 Months	6 Months	12 Months
Lindhe (1984) [26]	Adult and Juvenile Patients with Angular Bony Defects (Molar/Incisior Sites)	Tetracycline Administration, SRP, Surgical Elimination of Granulation Tissue with MWF	Tetracycline: 250 mg QID for 14 days	Up to 60 months	28	Adult periodontitis group	12	PD (mm)	6	-	3.25	3.38
								CAL (mm)	-	-	2.6	2.65
								BOP (%)	100	-	5	10
								Plaque (%)	100	-	14	12
						Juvenile periodontitis group	16	PD (mm)	7.95	-	3.55	3.45
								CAL (mm)	-	-	3.65	3.58
								BOP (%)	100	-	9	13
								Plaque (%)	67	-	19	23
Christersson (1985) [32]	Localized Juvenile Periodontitis	SRP Alone, SRP with MWF or Soft Tissue Curettage	NA	16 Weeks	25 lesions in 7 patients	Scaling and root planing alone	8 lesions in 3 patients	PD (mm)	8.3	-	-	-
								BOP (%)	100	70	-	-
								Plaque (%)	90	20	-	-
						Widman flap surgery	8 lesions in 4 patients	PD (mm)	6.3	4.4	-	-
								BOP (%)	100	70	-	-
								Plaque (%)	90	20	-	-
						Soft tissue curettage	9 lesions in 4 patients	PD (mm)	7.4	4.8	-	-
								BOP (%)	100	70	-	-
								Plaque (%)	90	20	-	-
Wennström (1986) [27]	Advanced Periodontal Disease	Split-Mouth: SRP Only or SRP + MWF	NA	5 Years	16 patients (106 sites)	Juvenile periodontitis patients	Surgical: 11 patients, 29 sites	PD (mm)	7.38 ± 1.18	-	3.71 ± 0.95	-
								CAL (mm)	-	-	2.24 ± 0.66	-
								BOP (%)	100	-	21	-
								Plaque (%)	79	-	10	-
							Non-surgical: 11 patients, 32 sites	PD (mm)	6.98 ± 0.89	-	3.81 ± 0.96	-
								CAL (mm)	-	-	2.01 ± 0.55	-
								BOP (%)	100	-	13	-
								Plaque (%)	72	-	9	-
						Post-juvenile periodontitis patients	Surgical: 5 patients, 22 sites	PD (mm)	6.5 ± 0.80	-	2.36 ± 0.50	-
								CAL (mm)	-	-	2.86 ± 0.94	-
								BOP (%)	100	-	9	-
								Plaque (%)	68	-	5	-
							Non-surgical: 5 patients, 23 sites	PD (mm)	6.68 ± 0.86	-	2.8 ± 0.84	-
								CAL (mm)	-	-	2.38 ± 0.65	-
								BOP (%)	100	-	9	-
								Plaque (%)	70	-	9	-
Mandell (1988) [36]	Localized Juvenile Periodontitis	OFD, Administration of Doxycycline	Doxycycline: 100 mg every 12 h on Day 1, 100 mg Daily for 14 days after	12 Months	8 patients, over 1200 sites	All patients had same treatment	8	PD (mm)	7.6 ± 0.4	3.8 ± 0.3	-	4.0 ± 0.4
								CAL (mm)	5.8 ± 0.4	4.6 ± 0.4	-	4.5 ± 0.4
								BOP (%)	95	27	-	32
								Gingival Redness (%)	77	23	-	18
Sewon (1992) [28]	Juvenile Periodontitis Patients	SRP, MWF as necessary	NA	65 Months	11 patients in the study, 10 received surgery therapy	All patients had same treatment	11	Plaque Index from Ramfjord’s Teeth	0.57 ± 0.37	-	-	0.23 ± 0.26
								Gingival Index from All Teeth	0.59 ± 0.58	-	-	0.15 ± 0.37
Buchmann (2002) [29]	Aggressive Periodontitis	SRP, MWF, Administration of AMOX and MET	Amoxicillin: 500 mg TID for 7 days; Metronidazole: 250 mg TID for 7 days	5 Years	13 patients	All patients had same treatment	13	CAL (mm)	7.85	5.62	5.57	5.71
Cirino (2019) [33]	Generalized Aggressive Periodontitis (GAP)	Initial Phase Therapy, then Split-Mouth: SRP or OFD	NA	12 Months	16 patients started, ended with 8	Surgical group	16	PD (mm)	6.2 ± 1.3	4.1 ± 0.7	4.2 ± 0.9	4.2 ± 0.7
								CAL (mm)	6.4 ± 1.3	5.2 ± 1.3	5.4 ± 1.6	5.1 ± 1.6
								BOP (%)	92.3	46.2	61.5	66.7
								Plaque (%)	46.2	46.2	46.2	37.5
						Non-surgical group	16	PD (mm)	6.2 ± 0.7	4.4 ± 0.7	4.4 ± 0.8	4.5 ± 0.6
								CAL (mm)	6.4 ± 0.8	4.9 ± 0.8	5.0 ± 1.0	5.2 ± 0.8
								BOP (%)	92.3	53.8	61.5	66.7
								Plaque (%)	46.2	46.2	53.8	50

**Table 3:** Summary studies using guided tissue regeneration in patients with molar–incisor pattern periodontitis.

Study Author (Year)	Type of Patient	Treatment Modality	Antibiotic	Duration	Total Number of Patients	Number of Patients per Group	Treatment Groups	Clinical Data									
								Variable	Baseline	After Baseline							
										3 Months	6 Months	9 Months	12 Months	18 Months	24 Months	36 Months	Up to 10 Years
**EMD**																	
Vandana (2004) [45]	4 CP patients and 4 AgP patients	Non-surgical therapy, occlusal adjustment, surgical debridement alone or surgical debridement with EMD	Post-surgical amoxicillin 250 mg, three times daily for 5 days	9 months	8	4, split-mouth	Surgical debridement	PD (mm)	6.75 ± 1.58	n/a	n/a	3.25 ± 1.75	n/a	n/a	n/a	n/a	n/a
								CAL (mm)	6.13 ± 2.85	n/a	n/a	2.75 ± 3.28	n/a	n/a	n/a	n/a	n/a
						4, split-mouth	Surgical debridement with EMD	PD (mm)	7.63 ± 1.06	n/a	n/a	4.5 ± 2.00	n/a	n/a	n/a	n/a	n/a
								CAL (mm)	6.50 ± 1.31	n/a	n/a	3.13 ± 2.17	n/a	n/a	n/a	n/a	n/a
Miliauskaite (2007) [46]	19-year-old LAgP patient	Non-surgical therapy, systemic antibiotics, papilla preservation technique and application of EMD or EMD and bioactive glass	Pre-surgical metronidazole (no dosage described)	Up to 3 years	1	1	Bioactive glass and EMD	PD (mm)	8.1 ± 1.7	n/a	2.8 ± 0.5	n/a	2.9 ± 0.7	n/a	3.0 ± 1.7	3.0 ± 0.6	n/a
								GR (mm)	3.0 ± 0.6	n/a	0.4 ± 0.6	n/a	0.4 ± 0.7	n/a	0.4 ± 0.7	0.5 ± 0.8	n/a
								CAL (mm)	8.6 ± 2.4	n/a	3.2 ± 0.7	n/a	3.3 ± 0.4	n/a	3.5 ± 0.7	3.7 ± 0.8	n/a
							EMD	PD (mm)	7.6 ± 2.0	n/a	2.4 ± 1.3	n/a	2.5 ± 1.6	n/a	2.5 ± 1.7	2.6 ± 1.5	n/a
								GR (mm)	.06 ± 1.5	n/a	1.0 ± 0.5	n/a	1.1 ± 0.4	n/a	1.1 ± 0.7	1.3 ± 2.3	n/a
								CAL (mm)	8.3 ± 3.2	n/a	3.5 ± 1.7	n/a	3.6 ± 2.8	n/a	3.6 ± 1.7	4.0 ± 3.6	n/a
Kaner (2009) [47]	27-year-old AgP patient.	Non-surgical therapy, systemic abx, minimally invasive flap surgery, EMD	Pre-surgical amoxicillin 500 mg, Ratiopharm, and metronidazole 250 mg, Artesan, both three times a day for 10 days	12 months	1	1, 4 sites per group	Flap and EMD	CAL (mm average of 4 sites)	10.5	n/a	n/a	n/a	n/a	4.25	n/a	n/a	n/a
								PD (mm average of 4 sites)	10.25	n/a	n/a	n/a	n/a	3	n/a	n/a	n/a
Artzi (2015) [48]	32 healthy AgP patients aged 14–25 with multiple intrabony defects	Non-surgical therapy, systemic abx, EMD and GTR, GTR	Pre-surgical amoxicillin 500 mg and metronidazole 250 mg (TID) for 1 week	12 months	32	16	GTR	PD (mm)	8.93 ± 1.14	n/a	n/a	n/a	3.58 ± 0.50	n/a	n/a	n/a	n/a
								CAL (mm)	9.03 ± 1.03	n/a	n/a	n/a	4.16 ± 0.53	n/a	n/a	n/a	n/a
							GTR with EMD	PD (mm)	8.77 ± 1.04	n/a	n/a	n/a	3.61 ± 0.36	n/a	n/a	n/a	n/a
								CAL (mm)	8.79 ± 1.04	n/a	n/a	n/a	3.77 ± 0.22	n/a	n/a	n/a	n/a
Artzi (2019) [49]	28 young, healthy AgP patients	Non-surgical therapy, systemic abx, surgical therapy of GTR or demineralized bone xenograft particles with EMD	Pre-surgical amoxicillin 500 mg + metronidazole 250 mg (TID) for a week	Up to 10 years	18	6 (12 surgical sites)	GTR	PD (mm)	6.23 ± 1.24	n/a	n/a	n/a	n/a	n/a	n/a	n/a	3.87 ± 1.02
								CAL (mm)	6.37 ± 1.37	n/a	n/a	n/a	n/a	n/a	n/a	n/a	4.1 ± 1.06
						12 (54 surgical sites)	EMD/DBX	PD (mm)	5.58 ± 1.34	n/a	n/a	n/a	n/a	n/a	n/a	n/a	3.64 ± 1.35
								CAL (mm)	6.16 ± 1.52	n/a	n/a	n/a	n/a	n/a	n/a	n/a	4.24 ± 1.30
Mazzonetto (2021) [50]	40 healthy AgP patients and 20 healthy CP patients	Non-surgical therapy, conservative surgery (CS) or CS with EMD	None	12 months	60 patients, 60 sites	20 patients, 20 sites	AgP and CS/EMD	PD (mm)	6.7 ± 1.1	n/a	4.7 ± 0.9	n/a	4.3 ± 0.9	n/a	n/a	n/a	n/a
								CAL (mm)	10.3 ± 1.8	n/a	8.3 ± 1.6	n/a	7.8 ± 1.5	n/a	n/a	n/a	n/a
								GR (mm)	0.7 ± 0.9	n/a	1.1 ± 1.3	n/a	0.7 ± 1.0	n/a	n/a	n/a	n/a
						20 patients, 20 sites	AgP and CS	PD (mm)	6.7 ± 1.1	n/a	4.9 ± 1.0	n/a	4.8 ± 1.0	n/a	n/a	n/a	n/a
								CAL (mm)	10.5 ± 1.4	n/a	9.3 ± 2.1	n/a	8.8 ± 1.6	n/a	n/a	n/a	n/a
								GR (mm)	0.8 ± 1.0	n/a	1.6 ± 1.3	n/a	1.1 ± 0.9	n/a	n/a	n/a	n/a
						20 patients, 20 sites	CP + CS/EMD	PD (mm)	6.6 ± 1.3	n/a	4.4 ± 1.4	n/a	4.2 ± 1.4	n/a	n/a	n/a	n/a
								CAL (mm)	11.3 ± 2.2	n/a	9.3 ± 2.3	n/a	9.2 ± 2.2	n/a	n/a	n/a	n/a
								GR (mm)	1.3 ± 1.0	n/a	1.6 ± 0.9	n/a	1.7 ± 0.9	n/a	n/a	n/a	n/a
**PRP**																	
Kang (2010) [51]	12 patients, 9 CP patients and 3 AgP patients with 15 defects	Non-surgical therapy, DFDBA or DFDBA with PRP	None	6 months	12	10	DFDBA	PI	1.7	n/a	1.5	n/a	n/a	n/a	n/a	n/a	n/a
								BI	2.7	n/a	1.8	n/a	n/a	n/a	n/a	n/a	n/a
								PD (mm)	6	n/a	3.9	n/a	n/a	n/a	n/a	n/a	n/a
								CAL (mm)	7	n/a	4.4	n/a	n/a	n/a	n/a	n/a	n/a
						5	DFDBA with PRP	BI	2.9	n/a	1.7	n/a	n/a	n/a	n/a	n/a	n/a
								PD (mm)	6.2	n/a	3.2	n/a	n/a	n/a	n/a	n/a	n/a
								CAL (mm)	7.1	n/a	3.6	n/a	n/a	n/a	n/a	n/a	n/a
Gupta (2014) [52]	10 AgP patients with bilateral intrabony defects	Non-surgical therapy, OFD and HA graft or OFD and HA graft with PRP	Post-surgical amoxicillin 500 mg every 6 h for 5 postoperative days	12 months	10	5	Mx OFD and HA graft	Plaque control	33.75 ± 12.9	17.5 ± 8.14	n/a	n/a	16.25 ± 5.59	n/a	n/a	n/a	n/a
								BOP	26.25 ± 6.84	16.25 ± 5.59	n/a	n/a	15 ± 10.458	n/a	n/a	n/a	n/a
								PD (mm)	6.2 ± 1.87	4.6 ± 0.96	n/a	n/a	4.3 ± 0.87	n/a	n/a	n/a	n/a
								RAL (relative attachment lvl)	13.5 ± 1.84	12.9 ± 0.87	n/a	n/a	12.3 ± 0.94	n/a	n/a	n/a	n/a
								Radiographic measurements	93.3 ± 30.8	n/a	n/a	n/a	66.0 ± 15.3	n/a	n/a	n/a	n/a
							Mx OFD and HA graft and PRP	Plaque control	25 ± 4.41	15 ± 7.12	n/a	n/a	13.75 ± 8.14	n/a	n/a	n/a	n/a
								BOP Mx	22.5 ± 10.45	22.5 ± 7.12	n/a	n/a	15 ± 13.69	n/a	n/a	n/a	n/a
								PD (mm)	6 ± 2.05	3.1 ± 0.87	n/a	n/a	2.8 ± 0.63	n/a	n/a	n/a	n/a
								RAL	13.9 ± 2.54	11.5 ± 1.77	n/a	n/a	10.9 ± 1.52	n/a	n/a	n/a	n/a
								Radiographic measurements	108.3 ± 37	n/a	n/a	n/a	19.76 ± 15.11	n/a	n/a	n/a	n/a
						5	Md OFD and HA graft	Plaque control Md control group	32.5 ± 9.27	18.75 ± 10.8	n/a	n/a	18.75 ± 6.25	n/a	n/a	n/a	n/a
								BOP	21.25 ± 8.38	17.5 ± 6.84	n/a	n/a	16.25 ± 5.59	n/a	n/a	n/a	n/a
								PD (mm)	6.6 ± 2.45	5.2 ± 1.22	n/a	n/a	4.7 ± 1.16	n/a	n/a	n/a	n/a
								RAL	14.7 ± 2.45	14 ± 1.41	n/a	n/a	13.3 ± 1.15	n/a	n/a	n/a	n/a
								Radiographic measurements	111.38 ± 49.35	n/a	n/a	n/a	88.53 ± 42	n/a	n/a	n/a	n/a
							Md OFD and HA graft and PRP	Plaque control	27.5 ± 7.12	13.75 ± 6.84	n/a	n/a	12.5 ± 8.83	n/a	n/a	n/a	n/a
								BOP Md	18.75 ± 6.25	15 ± 5.59	n/a	n/a	11.25 ± 2.79	n/a	n/a	n/a	n/a
								PD (mm)	6.6 ± 2.41	3.6 ± 0.69	n/a	n/a	3 ± 0.81	n/a	n/a	n/a	n/a
								RAL	14.6 ± 2.67	12.2 ± 1.47	n/a	n/a	11.5 ± 1.64	n/a	n/a	n/a	n/a
								Radiographic measurements	112.24 ± 48.40	n/a	n/a	n/a	16.6 ± 8.25	n/a	n/a	n/a	n/a
**PRF**																	
Kumar (2017) [53]	15 LAgP patients with a total of 30 sites, 2 sites per individual	Non-surgical therapy, systemic abx, modified flap operation (MFO) or MFO with autologous PRF	Pre-surgical amoxicillin 500 mg and metronidazole 400 mg 3 times a day for 7 days. Post-surgical amoxicillin 500 mg, metronidazole 400 mg, and ibuprofen 400 mg three times daily for 1 week	12 months	15	15, split-mouth	MFO and PRP	PD (mm)	8.17 ± 1.47	n/a	n/a	n/a	4.17 ± 1.16	n/a	n/a	n/a	n/a
								CAL (mm)	8.33 ± 0.81	n/a	n/a	n/a	4.33 ± 0.51	n/a	n/a	n/a	n/a
								GML (mm)	0.51 ± 0.54	n/a	n/a	n/a	0.33 ± 0.516	n/a	n/a	n/a	n/a
						15, split-mouth	MFO	PD (mm)	7.83 ± 1.47	n/a	n/a	n/a	6.33 ± 1.75	n/a	n/a	n/a	n/a
								CAL (mm)	7.17 ± 1.47	n/a	n/a	n/a	6.83 ± 1.94	n/a	n/a	n/a	n/a
								GML (mm)	0.47 ± 0.54	n/a	n/a	n/a	0.67 ± 0.51	n/a	n/a	n/a	n/a
Bajaj (2017) [54]	17 GAgP patients with 54 IBDs	Non-surgical therapy, open flap debridement or open flap debridement with PRF	Post-surgical amoxicillin 500 mg four times daily for 5 days	9 months	17	8	Control	PD (mm)	7.96 ± 1.28	n/a	n/a	5.81 ± 1.07	n/a	n/a	n/a	n/a	n/a
								CAL (mm)	6.74 ± 0.81	n/a	n/a	5.14 ± 0.86	n/a	n/a	n/a	n/a	n/a
								IBD depth(mm)	4.71 ± 0.71	n/a	n/a	3.86 ± 0.54	n/a	n/a	n/a	n/a	n/a
						9	Test	PD (mm)	8.03 ± 1.19	n/a	n/a	4.88 ± 1.05	n/a	n/a	n/a	n/a	n/a
								CAL (mm)	6.77 ± 0.84	n/a	n/a	4.11 ± 0.75	n/a	n/a	n/a	n/a	n/a
								IBD depth (mm)	4.81 ± 0.51	n/a	n/a	2.57 ± 0.49	n/a	n/a	n/a	n/a	n/a
**GTR**																	
Mengel (2006) [39]	16 GAgP patients (11 women and five men) with 1- to 3-walled intrabony defects with a depth of 4 mm and with preoperative probing depths (PDs) of 7 mm	Non-surgical therapy, GTR with membrane and GTR with bioactive glass	None	5 years	16 patients, 42 defects	22 defects	GTR with membrane	PI	0.7 ± 0.08	n/a	n/a	n/a	n/a	n/a	n/a	n/a	n/a
								GI	0.6 ± 0.07	n/a	n/a	n/a	n/a	n/a	n/a	n/a	n/a
								PD (mm)	7.7 ± 1.3	n/a	n/a	n/a	n/a	n/a	n/a	n/a	4.1 ± 1.6
								CAL (mm)	10.1 ± 2.1	n/a	n/a	n/a	n/a	n/a	n/a	n/a	7.1 ± 1.9
								GR (mm)	2.4 ± 2.2	n/a	n/a	n/a	n/a	n/a	n/a	n/a	1.8 ± 1.4
								TM	1.0 ± 1.1	n/a	n/a	n/a	n/a	n/a	n/a	n/a	n/a
						20 defects	GTR with bioactive glass	PI	0.5 ± 0.06	n/a	n/a	n/a	n/a	n/a	n/a	n/a	n/a
								GI	0.9 ± 0.08	n/a	n/a	n/a	n/a	n/a	n/a	n/a	n/a
								PD (mm)	7.5 ± 0.08	n/a	n/a	n/a	n/a	n/a	n/a	n/a	n/a
								CAL (mm)	9.8 ± 1.9	n/a	n/a	n/a	n/a	n/a	n/a	n/a	4.2 ± 1.8
								GR (mm)	2.4 ± 1.9	n/a	n/a	n/a	n/a	n/a	n/a	n/a	6.3 ± 2.7
								TM	0.8 ± 0.08	n/a	n/a	n/a	n/a	n/a	n/a	n/a	2.2 ± 1.7
Queiroz (2013) [40]	15 GAgP patients, with two intrabony defects ≥3 mm deep	Non-surgical therapy, ABM/P−15 or GTR	Pre-surgical amoxicillin 500 mg with metronidazoe 24 days before the scaling	6 months	15 patients	15 patients, split-mouth	ABM/P−15	PD (mm)	5.36 ± 1.28	n/a	3.09 ± 0.84	n/a	n/a	n/a	n/a	n/a	n/a
								CAL (mm)	10.31 ± 1.29	n/a	8.45 ± 1.18	n/a	n/a	n/a	n/a	n/a	n/a
								GR (mm)	0.27 ± 0.44	n/a	0.86 ± 0.54	n/a	n/a	n/a	n/a	n/a	n/a
								rCEJ-rAC (mm)	2.82 ± 1.21	n/a	3.14 ± 1.63	n/a	n/a	n/a	n/a	n/a	n/a
								rCEJ-rBD (mm)	6.07 ± 1.72	n/a	3.58 ± 1.45	n/a	n/a	n/a	n/a	n/a	n/a
						15 patients, split-mouth	GTR	PD (mm)	5.41 ± 1.23	n/a	2.85 ± 0.50	n/a	n/a	n/a	n/a	n/a	n/a
								CAL (mm)	11.15 ± 1.28	n/a	9.05 ± 1.43	n/a	n/a	n/a	n/a	n/a	n/a
								GR (mm)	0.29 ± 0.48	n/a	0.92 ± 0.71	n/a	n/a	n/a	n/a	n/a	n/a
								rCEJ-rAC (mm)	3.57 ± 1.10	n/a	4.24 ± 1.08	n/a	n/a	n/a	n/a	n/a	n/a
								rCEJ-rBD (mm)	6.22 ± 1.59	n/a	5.48 ± 1.02	n/a	n/a	n/a	n/a	n/a	n/a
Rakmanee (2016) [37]	18 AgP patients who had similar bilateral intrabony defects	SPPF alone or with the placement of a resorbable GTR membrane	none	12 months	18	18, split-mouth	AF	CEJ-BD distance (mm)	7.2 ± 1.0	n/a	5.9 ± 0.4	n/a	5.6 ± 0.8	n/a	n/a	n/a	n/a
								Defect depth (mm)	5.1 ± 0.6	n/a	3.4 ± 0.7	n/a	2.9 ± 0.6	n/a	n/a	n/a	n/a
								CEJ-BC distance (mm)	2.2 ± 0.8	n/a	2.6 ± 0.7	n/a	2.7 ± 1.0	n/a	n/a	n/a	n/a
						18, split-mouth	GTR	CEJ-BD distance (mm)	8.2 ± 1.3	n/a	6.6 ± 1.2	n/a	6.4 ± 1.1	n/a	n/a	n/a	n/a
								Defect depth (mm)	6.0 ± 1.0	n/a	3.3 ± 0.8	n/a	3.4 ± 0.7	n/a	n/a	n/a	n/a
								CEJ-BC distance (mm)	2.2 ± 0.8	n/a	3.3 ± 1.1	n/a	3.1 ± 1.2	n/a	n/a	n/a	n/a
Rakmanee (2016) [38]	18 AgP patients with similar bilateral intrabony defects	Non-surgical therapy, polyglycolide membrane according to the GTR principle versus access flap surgery	Post-surgical antibiotics were prescribed to the patients with membrane exposures. Metronidazole 400 mg three times/day for 2 weeks	12 months	18	18, split-mouth	Access flap (AF)	CAL (mm)	6.6 ± 0.3	n/a	4.9	n/a	4.4	n/a	n/a	n/a	n/a
								PPD (mm)	6.1 ± 0.4	n/a	4.2	n/a	3.6	n/a	n/a	n/a	n/a
						18, split-mouth	Polyglycolide membrane according to GTR	CAL (mm)	6.5 ± 0.4	n/a	5	n/a	5.2	n/a	n/a	n/a	n/a
								PPD (mm)	6.5 ± 0.4	n/a	3.8	n/a	3.9	n/a	n/a	n/a	n/a
Gorski (2020) [41]	15 AgP patients, two deep intrabony defects	Non-surgical therapy, xenogenic graft plus modified perforated membranes or xenogenic graft plus standard collagen membrane	Pre-surgical amoxicillin 500 mg and metronidazole 250 mg three times a day for 1 week	4 years	15	15, splitmouth	Deproteinized bovine bone mineral (DBBM) plus modified perforated membranes (MPMs)	PD (mm)	7.4 ± 1.5	n/a	n/a	n/a	3.4 ± 1.1	n/a	n/a	n/a	3.6 ± 1.3
								CAL (mm)	8.7 ± 1.6	n/a	n/a	n/a	4.0 ± 1.6	n/a	n/a	n/a	4.0 ± 1.7
								GR (mm)	1.3 ± 0.7	n/a	n/a	n/a	0.9 ± 1.1	n/a	n/a	n/a	0.6 ± 0.8
								DD (mm)	5.9 ± 1.2	n/a	n/a	n/a	0.7 ± 0.9	n/a	n/a	n/a	0.6 ± 0.5
						15, splitmouth	DBBM plus standard collagen membranes (CMs)	PD (mm)	7.2 ± 1.3	n/a	n/a	n/a	3.7 ± 0.9	n/a	n/a	n/a	3.8 ± 1.0
								CAL (mm)	8.5 ± 1.8	n/a	n/a	n/a	4.2 ± 1.2	n/a	n/a	n/a	4.6 ± 1.1
								GR (mm)	1.5 ± 1.1	n/a	n/a	n/a	1.5 ± 2.2	n/a	n/a	n/a	0.9 ± 1.1
								DD (mm)	5.3 ± 1.8	n/a	n/a	n/a	0.9 ± 0.6	n/a	n/a	n/a	0.7 ± 0.4

**Table 4:** Summary studies using laser and antimicrobial photodynamic therapy in patients with molar–incisor pattern periodontitis.

Study Author (Year)	Type of Patient	Treatment Modality	Antibiotic Regimen (If Administered)	Treatment Groups	Duration	Number of Patients per Group	Clinical Data	Baseline	After Baseline			
									Retreatment or 1 Month	1–3 Months	4–6 Months	24 Months
Talmac (2019) [73]	Generalized Aggressive Periodontitis	All individuals received non-surgical periodontal treatment. SRP (with control and experimental) in 2 visits. Diode laser and Er,Cr:YSGG laser treatments were conducted after SRP. The procedures applied to all individuals in the study in 4 different quadrants as a “split-mouth” study	N/A	Control (SRP)	2 sessions	26	PD (mm)	3.91 ± 0.6		3.34 ± 0.4		
							CAL (mm)	4.5 ± 0.93		3.9 ± 0.86		
							BOP (%)	50 ± 18.7		28.84 ± 13		
							PI	1.51 ± 0.2		1.26 ± 13		
				SRP + Diode Laser (iLase)	2 sessions	26	PD (mm)	3.99 ± 0.76		3.28 ± 0.6		
							CAL (mm)	4.4 ± 0.76		3.69 ± 0.6		
							BOP (%)	51.92 ± 19.9		26.92 ± 19		
							PI	1.54 ± 0.19		1.23 ± 0.2		
				SRP + Er,Cr:YSGG Laser (Waterlase)	2 sessions	26	PD (mm)	3.96 ± 0.59		3.15 ± 0.4		
							CAL (mm)	4.56 ± 1.04		3.71 ± 0.7		
							BOP (%)	53.84 ± 23.12		15.38 ± 15		
							PI	1.53 ± 0.25		1.13 ± 0.1		
Ertugrul (2017) [74]	Generalized Aggressive Periodontitis	All individuals received non-surgical periodontal treatment. SRP (with control and experimental) in 2 visits. Diode laser and Er,Cr:YSGG laser treatments were conducted after SRP. The procedures applied to all individuals in the study in 4 different quadrants as a “split-mouth” study	N/A	Control (SRP) for Diode	2 sessions	13	PD (mm)	6 ± 0.64	4.78 ± 0.32			
							CAL (mm)	6.12 ± 0.74	4.67 ± 0.22			
							BOP (%)	100	41 ± 6			
							PI	1.66 ± 0.14	0.44 ± 0.09			
				SRP + Diode Laser (iLase)	2 sessions	13	PD (mm)	6.14 ± 0.54	4.28 ± 0.39			
							CAL (mm)	6 ± 0.51	4.11 ± 0.38			
							BOP (%)	100	56.97 ± 26			
							PI	1.51 ± 0.41	0.42 ± 0.09			
				Control (SRP) for Er,Cr:YSGG Laser (Waterlase)	2 sessions	13	PD (mm)	6.23 ± 0.91	4.78 ± 0.61			
							CAL (mm)	5.45 ± 0.89	3.34 ± 0.78			
							BOP (%)	100	56.97 ± 26			
							PI	1.62 ± 0.22	0.35 ± 0.05			
				SRP + Er,Cr:YSGG Laser (Waterlase)	2 sessions	13	PD (mm)	5.82 ± 0.94	3.99 ± 1.12			
							CAL (mm)	5.13 ± 0.95	3.56 ± 1.1			
							BOP (%)	88.85 ± 1.1	50 ± 6			
							PI	1.73 ± 0.19	0.32 ± 0.04			
Annaji (2016) [72]	Localized/Generalized Aggressive Periodontitis (Age 18–35)	**Quadrant 1 (Q1):** SRP alone with ultrasonic scaler **Quadrant 2 (Q2):** SRP + Laser irradiation with 810 nm at 1 W, continuous mode for 30 seconds per tooth (AMD Picasso^®^ DENTSPLY India Pvt. Ltd., India) **Quadrant 3 (Q3):** SRP + Photodynamic Therapy (PDT) on “0” day (Toluidine blue—O dye 1 mg/mL and diode laser 810 nm) **Quadrant 4 (Q4):** SRP + PDT on 0, 7th, and 21st day (Toluidine blue—O dye 1 mg/mL and diode laser 810 nm)	N/A	Control (SRP) Q1	1 session	15	PD (mm)	6.08 ± 0.21		5.79 ± 0.2		
							CAL (mm)	9.41 ± 0.51		9.13 ± 0.46		
							BOP (# of site)	3.16 ± 0.35		1.72 ± 0.2		
							PI	2.61 ± 0.18		1.58 ± 0.26		
				SRP + Diode Laser (Picasso) Q2	1 session	15	PD (mm)	6.13 ± 0.35		5.79 ± 0.2		
							CAL (mm)	9.63 ± 0.77		9.17 ± 0.84		
							BOP (# of site)	3.28 ± 0.35		1.58 ± 0.23		
							PI	2.51 ± 0.2		1.59 ± 0.25		
				SRP + Diode (Picasso) + PDT (only day0) Q3	1 session	15	PD (mm)	6.2 ± 0.25		5.59 ± 0.47		
							CAL (mm)	9.52 ± 0.58		8.87 ± 0.71		
							BOP (# of site)	3.27 ± 1.57		1.57 ± 0.23		
							PI	2.53 ± 0.21		1.51 ± 0.29		
				SRP + Diode (Picasso) + PDT (0,7,21 day) Q4	3 sessions	15	PD (mm)	6.21 ± 0.27		5.4 ± 0.47		
							CAL (mm)	9.93 ± 0.43		8.63 ± 0.61		
							BOP (# of site)	3.27 ± 0.32		1.39 ± 0.17		
							PI	2.54 ± 0.21		1.51 ± 0.29		
Matarese (2017) [66]	Generalized Aggressive Periodontitis	The maxillary right and left quadrants were randomly assigned to SRP + Diode Laser or SRP alone	N/A	Control (SRP)	1 session	31	PD (mm)	5.18 ± 0.57	4.32 ± 0.49	4.26 ± 0.28	2.68 ± 0.29	3.36 ± 0.51
							CAL (mm)	4.88 ± 0.55	4.79 ± 0.44	4.84 ± 0.42	3.11 ± 0.25	4.23 ± 0.21
							BOP (%)	78.12 ± 2.2	38.32 ± 4.1	30.32 ± 2.8	24.67 ± 3.2	32.26 ± 3.1
							Plaque Score (%)	28.54 ± 5.23	26.84 ± 5.31	27.01 ± 4.68	25.04 ± 3.69	17.94 ± 3.24
				SRP + Diode Laser (Wiser Laser Doctor Smile)	1 session	31	PD (mm)	5.25 ± 0.66	3.79 ± 0.51	2.38 ± 0.25	2.24 ± 0.35	2.56 ± 0.44
							CAL (mm)	5.36 ± 0.39	4.66 ± 0.39	3.59 ± 0.41	3.19 ± 0.22	3.44 ± 0.28
							BOP (%)	74.26 ± 3.6	37.23 ± 4.2	29.38 ± 3.3	22.79 ± 4.2	26.16 ± 2.4
							Plaque Score (%)	28.21 ± 5.12	27.33 ± 4.85	26.12 ± 5.14	24.55 ± 3.36	18.27 ± 3.13
Kamma (2009) [75]	Generalized Aggressive Periodontitis (smokers included)	**Session 1:** Supragingival scaling only **Session 2 (2 wks later)**: Each quad was randomly treated. SRP alone, Diode Laser (980 nm) (LAS) treatment alone, and SRP combined with LAS (SRP + LAS). One quadrant was not treated and served as a control (CRL)	N/A	SRP	2 sessions	30	PD (mm)	6.47 ± 1.356		4.13 ± 1.06	4.13 ± 1.06	
							CAL (mm)	7.07 ± 1.58		5.27 ± 1.751	5.2 ± 1.656	
							BOP (# of site)	81.6		25.8		
							PI	54.1		32.6		
				SRP + LAS	2 sessions	30	PD (mm)	6.67 ± 1.291		3.8 ± 0.941	3.87 ± 0.915	
							CAL (mm)	7.07 ± 1.71		4.93 ± 1.668	4.93 ± 1.668	
							BOP (# of site)	82.4		24.3		
							PI	52.7		29.2		
				LAS	2 sessions	30	PD (mm)	5.93 ± 1.163		3.93 ± 1.387	3.93 ± 1.387	
							CAL (mm)	6.87 ± 1.598		4.93 ± 1.668	4.93 ± 1.668	
							BOP (# of site)	83.7		23.2		
							PI	50.7		31.6		
				CTRL (supragingival prophylaxis)	1 session	30	PD (mm)	6.2 ± 1.74		6.07 ± 1.624	6.07 ± 1.624	
							CAL (mm)	7.00 ± 2.104		6.73 ± 1.71	6.73 ± 1.71	
							BOP (# of site)	90.8		35.4		
							PI	55.9		36.5		
Al-Khureif (2020) [68]	Generalized Aggressive Periodontitis	All patients in both groups received one-stage full-mouth SRP under local anesthetic with ultrasonic **PDT group:** SRP + HELBO Blue, HELBO Photodynamic Systems. The procedure was repeated at 3, 7, and 14 days of follow-up. **Antimicrobial group:** MTZ 500 mg + AMX500 mg TID for 7 days	7 days TID of 500 mg metronidazole 500 mg amoxicillin for the Antimicrobial group	SRP + aPDT (0, 3, 7, and 14 days, HELBO Photodynamic Systems, Diode laser)	4 sessions	9	Moderate PD (mm)	5.44 ± 0.39		3.23 ± 0.68	2.74 ± 0.46	
							Deep PD (mm)	7.63 ± 0.85		5.51 ± 1.23	3.82 ± 0.98	
							Moderate CAL (mm)	5.69 ± 0.84		3.27 ± 1.18	3.00 ± 0.94	
							Deep CAL (mm)	7.72 ± 1.18		5.03 ± 1.05	3.94 ± 1.01	
							BOP (%)	45.72 ± 7.6		23.61 ± 5.1	15.48 ± 4.9	
							PI	17.43 ± 3.2		8.63 ± 2.1	10.04 ± 2.6	
				SRP + MTZ/AMX	1 session + 7 days	8	Moderate PD (mm)	5.61 ± 0.35		3.71 ± 0.76	2.95 ± 0.45	
							Deep PD (mm)	7.81 ± 0.8		6.12 ± 1.29	4.85 ± 0.85	
							Moderate CAL (mm)	5.74 ± 0.89		4.06 ± 1.07	3.15 ± 1.04	
							Deep CAL (mm)	7.93 ± 0.77		6.25 ± 1.33	5.75 ± 0.56	
							BOP (%)	36.83 ± 9.5		19.68 ± 7.3	17.91 ± 6.8	
							PI	21.78 ± 5.8		12.44 ± 3.6	16.53 ± 4.9	
Oliveria (2007) [61]	Generalized Aggressive Periodontitis	**Group A**: SRP with hand instruments only **Group B**: aPDT only	N/A	SRP	1 session	10	PD (mm)	4.92 ± 1.14		3.98 ± 1.76		
							Deepest PD (mm)					
							CAL (mm)	10.53 ± 2.3		9.01 ± 3.05		
							BOP (%)	60		21		
							PI	1 ± 0.5		0.6 ± 0.4		
				aPDT (Helbo Therapielaser, Diode laser)	1 session	10	PD (mm)	4.92 ± 1.61		3.49 ± 0.98		
							CAL (mm)	9.93 ± 2.1		8.74 ± 2.12		
							BOP (%)	57		19		
							PI	1 ± 0.5		0.7 ± 0.4		
Arweiler (2014) [70]	Generalized Aggressive Periodontitis	All pockets ≥4 mm were treated by SRP using ultrasonic and hand instruments **PDT group:** HELBO^®^ Blue Photosensitizer with HELBO^®^ minilaser **Antibiotic group:** Prescribed 375 mg of amoxicillin and 250 mg of metronidazole TID for 7 days (starting on the day of SRP)	7 days TID of 250 mg metronidazole 375 mg amoxicillin per day for the Antibiotic group	SRP + aPDT (HELBO Photodynamic Systems, Diode laser)	2 sessions of aPDT	17	PD (mm)	5.1 ± 0.5			3.9 ± 0.8	
							PD ≥ 7 mm (mm)	7.7 ± 0.63			5.5 ± 1.26	
							CAL (mm)	5.7 ± 0.8			5.5 ± 1.1	
							CAL (PD ≥ 7 mm) (mm)	8.4 ± 1.2			6.4 ± 1.5	
							BOP (%)	70.4 ± 22.4			48.0 ± 22.2	
							PI	1.4 ± 0.7			0.5 ± 0.5	
				SRP + MTZ/AMX	1 session + 7 days	18	PD (mm)	5.0 ± 0.8			3.0 ± 0.6	
							PD ≥ 7 mm (mm)	7.6 ± 0.6			3.7 ± 0.9	
							CAL (mm)	5.5 ± 1.1			3.6 ± 0.9	
							CAL (PD ≥ 7 mm) (mm)	8.4 ± 1.1			4.6 ± 1.3	
							BOP (%)	85.7 ± 15.9			32.6 ± 21	
							PI	1.7 ± 0.8			0.4 ± 0.6	
Borekci (2007) [69]	Generalized Aggressive Periodontitis	**Control Group:** SRP with ultrasonic and hand instruments **Test Group:** Following SRP, toluidine blue O-mediated PDT was performed with an LED source	N/A	SRP	2 sessions	12	PD (mm)	4.92 ± 0.5		3.94 ± 0.52		
							PD ≥ 5 mm (mm)	6.19 ± 0.58		4.52 ± 0.71		
							CAL (mm)	8.8 ± 1.1		8.21 ± 1.14		
							CAL (PD ≥ 5 mm) (mm)	10.02 ± 1.2		8.81 ± 0.91		
							BOP (# of sites)	3.0 ± 0.9		0.91 ± 0.34		
							PI	2.0 ± 0.5		0.41 ± 0.13		
				SRP + aPDT (FotoSan, LED)	3 sessions	12	PD (mm)	4.56 ± 0.54		3.47 ± 0.52		
							PD ≥ 5 mm (mm)	6.25 ± 0.68		4.85 ± 0.97		
							CAL (mm)	8.62 ± 1.23		8.0 ± 1.02		
							CAL (PD ≥ 5 mm) (mm)	10.32 ± 1.13		9.2 ± 1.12		
							BOP (# of sites)	3.61 ± 0.63		0.83 ± 0.32		
							PI	2.41 ± 0.42		0.42 ± 0.11		

## Data Availability

Not applicable.
